# Sleep-related hypermotor epilepsy associated mutations uncover important kinetic roles of α4β2- nicotinic acetylcholine receptor intracellular structures

**DOI:** 10.1371/journal.pone.0247825

**Published:** 2021-03-03

**Authors:** Maegan M. Weltzin, Andrew A. George, Ronald J. Lukas, Paul Whiteaker

**Affiliations:** Division of Neurobiology, Barrow Neurological Institute, St. Joseph’s Hospital and Medical Center, Phoenix, Arizona, United States of America; Weizmann Institute of Science, ISRAEL

## Abstract

Sleep-related hypermotor epilepsy (SHE) is a group of seizure disorders prominently associated with mutations in nicotinic acetylcholine receptors (nAChR). The most prevalent central nervous system nAChR subtype contains α4 and β2 subunits, in two ratios. (α4β2)_2_β2-nAChR have high agonist sensitivity (HS-isoform), whereas (α4β2)_2_α4-nAChR agonist responses exhibit a small high-sensitivity, and a predominant low-sensitivity, phase of function (LS-isoform). Multiple non-synonymous mutations in the second and third transmembrane domains of α4 and β2 subunits are associated with SHE. We recently demonstrated that two additional, SHE-associated, missense mutations in the major cytoplasmic loops of these subunits [α4(R336H) and β2(V337G)] cause increased macroscopic function-per receptor. Here, we use single-channel patch-clamp electrophysiology to show that these mutations influence single-channel amplitudes and open- and closed-state kinetics. Pure populations of HS- or LS-isoform α4β2-nAChR were expressed by injecting either 1:10 or 30:1 α4:β2 cRNA ratios, respectively, into *Xenopus laevis* oocytes. Functional properties of the resulting mutant α4β2-nAChR isoforms were compared to their wildtype counterparts. α4(R336H) subunit incorporation minimally affected single-channel amplitudes, whereas β2(V337G) subunit incorporation reduced them significantly in both isoforms. However, for both mutant subunits, increased function-per-receptor was predominantly caused by altered single channel kinetics. The α4(R336H) mutation primarily destabilizes desensitized states between openings. By contrast, the β2(V337G) mutation principally stabilizes receptor open states. The use of naturally-occurring and physiologically-impactful mutations has allowed us to define valuable new insights regarding the functional roles of nAChR intracellular domains. Further mechanistic context is provided by intracellular-domain structures recently published for other members of the Cys-loop receptor superfamily (α3β4-nAChR and 5-HT_3A_R).

## Introduction

Sleep-related hypermotor epilepsy (SHE; previously named nocturnal frontal lobe epilepsy, NFLE) is a syndrome characterized by seizure onset mostly during sleep, rapid uncoordinated limb movements, and/or tonic-dystonic postures [1]. As noted by Tinuper and colleagues, SHE is of interest across a wide variety of clinical specializations due to difficulty in diagnosis and consequent issues with correct treatment. Discovery of an autosomal dominant form of SHE (ADSHE) was rapidly followed by discovery of the first causal gene, a missense mutant in CHRNA4 that encodes the α4 nicotinic acetylcholine receptor (nAChR) subunit [2,3]. Subsequent studies have shown that multiple mutations across nicotinic subunit genes CHRNA2, CHRNA4, and CHRNB2 are linked to ADSHE [1] and thus have made nAChR mutant subunits an area of particular interest.

α4β2*-nAChR (*denotes the possible presence of other subunits; [4]) are the most prevalent nAChR subtype expressed in the central nervous system [5]. Interest in this subtype is further heightened by the fact that eight distinct mutations of amino acids in the functionally critical second or third transmembrane (M2 and M3) domains, or the short linker between them, of nAChR α4 or β2 nAChR subunits are associated with ADSHE [3,6–11]. However, the M2 and M3 domains are not the only location in which SHE-associated mutations of the α4 or β2 nAChR subunits have been found. Two further examples were identified in the major intracellular domain that follows M3; one in each of the α4 and β2 subunits [12,13]. Our recent study was the first to characterize macroscopic functional effects of incorporating these SHE-associated α4(R336H) and β2(V337G) mutants into α4β2-nAChR [14]. As we and others have shown, α4β2-nAChR exist in two isoforms with distinct functional properties and subunit stoichiometries. (α4β2)_2_β2-nAChR have only high sensitivity to agonist activation, while agonist activation of (α4β2)_2_α4-nAChR typically exhibits a small high-sensitivity phase and a much larger low-sensitivity phase [15–21]. Accordingly, (α4β2)_2_β2- or (α4β2)_2_α4-nAChR are conventionally referred to as “high-sensitivity” (HS-isoform) or “low-sensitivity” (LS-isoform), respectively. The major finding of our prior study was that macroscopic functional effects of incorporating intracellular loop α4(R336H) and β2(V337G) mutant subunits share several key similarities with those previously determined for other SHE-associated mutations of residues in the transmembrane domains of α4 or β2 subunits. Specifically, these effects included an overall gain in α4β2-nAChR function without a significant change in cell-surface expression (and, therefore, a gain in function-per-receptor), and a bias towards HS-isoform expression in mixed populations of HS- and LS-isoform α4β2-nAChR. We proposed that these common effects, maintained across a large group of missense mutations in nAChR α4 and β2 subunits, may represent a macroscopic functional signature responsible for inducing SHE [14].

Receptor-level mechanisms underpinning these SHE-associated changes have, until now, not been studied definitively. A macroscopic study used a positive allosteric modulator (PAM) to infer that a set of three SHE-associated mutations located in the M2-M3 linker region of the transmembrane domain likely increases the open probability of α4β2-nAChR [22]. The α4(R336H) and β2(V337G) mutations are located within a portion of the large intracellular domain, between M3 and M4, that is known to influence conductance and desensitization rates [12,13,23,24]. We hypothesized, therefore, that changes in unitary amplitudes and closed-state kinetics caused by these mutations might explain (or be an important contributor to) the gain-of-function macroscopic-level effects that we previously observed [14]. These hypotheses are linked by a common theme; SHE-associated mutations exert their effects by causing specific receptor-level changes in the function of the nAChR subtypes that contain them. More recent work by us and others has identified unitary properties that are distinct for each α4β2-nAChR isoform [25,26]. This provided us, in the current study, the opportunity to probe the nature of these SHE-associated changes by comparison to the already-established properties of wildtype α4β2-nAChR isoforms. As we demonstrate, the single-channel functional effects of the α4(R336H) and β2(V337G) SHE-associated mutations are complex, but the previously observed macroscopic gain in function-per-receptor and bias towards HS-isoform function predominantly are explained by changes in single-channel closed and open kinetics, respectively.

These findings have both clinical and scientific importance. Because SHE has a low tendency for spontaneous remission and a relatively high incidence of resistance to antiepileptic drug treatment [27], better mechanistic understanding of how SHE-associated mutations exert their effects provides potential bases for discovering and developing improved therapeutic options for SHE and other focal epilepsies [1]. Further, insights gained from studying SHE likely will be beneficial as this disease provides a unique opportunity to understand focal epilepsy origins and pathogenesis [28,29]. Scientifically, compared to other regions of the receptor complex, the functional contributions of nAChR intracellular domains are largely understudied. Further, recent structural analysis of α4β2-nAChR does not provide information about this region [30]. However, the equivalent locations of the α4(R336H) and β2(V337G) residues are contained within the now-defined structures of two closely homologous members of the Cys-loop superfamily of ligand gated ion channels (LGICs), 5-hydroxytryptamine 3A receptor (5-HT_3A_R) and α3β4-nAChR [31,32]. The 5-HT_3A_R cryo-electron microscopy structures are especially informative here since they capture the architecture of the receptor in both closed and open states. As will be discussed, our enhanced understanding of the distinct functional effects of these disease-linked α4(R336H) and β2(V337G) nAChR mutations, therefore, offers significant new insights into the functional roles of nAChR intracellular domains.

## Materials and methods

### Reagents

Reagents were purchased from Sigma (St. Louis, MO, USA) unless specified otherwise, and fresh solution stocks were prepared daily, diluted, and filtered as required.

### DNA constructs and cRNA synthesis

#### Heterologous expression of human α4 and β2 nAChR subunits

As previously described [14], full-length cDNAs for wildtype human nAChR α4 and β2 subunits, as well as the SHE-associated α4(R336H) and β2(V337G) subunits (residue numbering begins at the methionine translation start), were synthesized and sequenced to confirm identity (Thermo Fisher Scientific, Waltham, MA, USA), before being ligated into the pCI mammalian expression vector (Promega Madison, WI, USA). Swa I linearized cDNA was used to synthesize cRNA using the mMessage mMachine T7 Transcription kit (Thermo Fisher Scientific). Purity was confirmed on a 1% agarose gel and final products were sub-aliquoted and stored at -80°C.

### Oocyte isolation and cRNA microinjections

*Xenopus laevis* harvested and de-folliculated stage V oocytes were purchased from EcoCyte Bioscience (Austin, TX, USA). cRNA microinjection and oocyte incubation conditions precisely followed methods described in previous studies [14,25,33]. Expression of HS- or LS-α4β2-nAChR isoforms containing the α4(R336H) SHE-associated mutant subunit was achieved in *Xenopus laevis* oocytes by injection of unlinked subunits at different cRNA subunit ratios (1 ng α4(R336H): 10 ng β2 to force expression of the HS-isoform (α4(R336H)β2)_2_β2-nAChR, or 30 ng α4(R336H): 1 ng β2 for LS-isoform (α4(R336H)β2)_2_α4(R336H)-nAChR). To express HS- or LS-α4β2-nAChR isoforms containing the β2(V337G) subunit, the same injection ratios were used as described immediately above except that the subunit cDNAs used encoded wildtype α4 and SHE-associated β2(V337G) subunits. Oocytes expressing the resulting HS- or LS-α4β2-nAChR isoforms harboring SHE mutant subunits were recorded from 3–6 days post cRNA injection. Our prior publications demonstrate that pure, uniform, populations of the intended α4β2-nAChR isoforms are expressed within this timeframe, as validated at the level of macroscopic, whole-cell, responses [14], and at the level of single-channel responses [25]. All constructs were tested using expression from at least three separately synthesized batches of cRNA, and at least three separate batches of oocytes.

### Single-channel patch-clamp electrophysiological recordings

As noted earlier, single-channel electrophysiological recordings from *Xenopus laevis* oocytes expressing α4β2-nAChR isoforms containing SHE-associated mutant subunits were obtained under the same conditions as, and in parallel to, those previously reported for wildtype α4β2-nAChR [25]. To summarize, oocyte vitelline membranes were removed using sharp forceps under a dissecting microscope (magnification = 20x total magnification). The stripped oocytes were transferred to a recording chamber containing oocyte Ringer’s solution (OR2; 92.5 mM NaCl, 2.5 mM KCl, 1 mM MgCl_2_∙6H_2_O, 1 mM CaCl_2_∙2H_2_O, and 5 mM HEPES; pH 7.5). Atropine sulfate (1.5 μM) was added to all recording and bath solutions to eliminate any potential muscarinic responses in response to ACh application. Patch pipettes were manufactured from thick-walled (2 mm outer diameter, 1.12 mm inner diameter) borosilicate glass capillary tubes (World Precision Instruments, Inc., Sarasota, FL, USA). Electrodes were fire-polished using a World Precision Instruments microforge (final resistance = 15–20 MΩ). Recordings were performed in cell-attached configuration (22°C) using an Axopatch 200B amplifier (Molecular Devices, Sunnyvale, CA, USA). Recordings were filtered on-line at 5 kHz, digitized at 50 kHz using an Axon Digidata 1550 (Molecular Devices), and stored on a personal computer for later analysis. Inward currents mediated by α4β2-nAChR containing SHE-associated mutant subunits were elicited with ACh (OR2 + ACh within the patch electrode; concentrations used are addressed in the next paragraph). Recordings used a +70 mV holding potential (corresponding to a transmembrane potential of approximately -100 mV). The presence of endogenous mechanosensitive channels was tested for by application of negative pressure to the patch, once formed. Data were discarded from any patches in which mechanosensitive events were observed, or in which seal resistance was < 8 GΩ. Under these conditions we observed no channel openings from oocytes expressing α4β2-nAChR isoforms, in the absence of ACh.

As shown in our previous publication, macroscopic EC_50_ values for ACh agonism of α4β2-nAChR are not changed by the inclusion of either α4(R336H) or β2(V337G) subunits, when compared to those measured for α4β2-nAChR containing only wildtype subunits. This is true for either of the HS- or LS-isoforms [14]. Accordingly, we were able to apply the same ACh concentrations to stimulate responses in this study of effects of α4(R336H) or β2(V337G) subunit incorporation as were used in our preceding single-channel study of wildtype α4β2-nAChR HS- and LS-isoform function [25]. Macroscopic HS-isoform α4β2-nAChR concentration-response curves are monophasic and, therefore, single-channel responses were stimulated using a single ACh concentration (1.3 μM ACh; corresponding to the macroscopic EC_50_). However, LS-isoform α4β2-nAChR macroscopic concentration responses display biphasic curves, having distinct HS- and LS-phases [14]. Therefore, we stimulated LS-isoform α4β2-nAChR responses in the presence of 0.7 μM ACh (HS-phase EC_50_; low concentration), or in the presence of 30 μM ACh (LS-phase EC_50_; high concentration), to activate either HS- or LS-phase responses. Applying ACh concentrations corresponding to macroscopic EC_50_ values of the α4β2-nAChR isoforms of interest ensured that sufficient openings were produced to allow efficient collection of data. It also avoided the risk of provoking either channel block by ACh at very high concentrations [34], or producing multiple, overlapping single-channel events.

### Single-channel patch-clamp electrophysiology data analysis

Using exactly the same approach to analysis as was previously applied [25], data recordings of single-channel responses were filtered off-line at 1 kHz and analyzed using the model-based-analysis program QuB [35]. To measure unitary amplitudes, open- and closed-dwell time durations, open probabilities, and burst properties, single-channel records were analyzed using the segmental K-means (SKM) idealization method. The maximum interval likelihood (MIL) feature was used to determine open- and closed-dwell time distributions [36,37]. The number of states used to best fit the open- and closed-dwell time histograms was determined by adding additional open or closed states to the model for each studied combination of receptor composition and ACh concentration. An optimal number of open and closed states was determined after the maximum likelihood estimation failed to improve the log likelihood (LL) score by > 10 units. Stability plots were generated for all recordings of α4β2-nAChR containing SHE mutant subunits, and were used to examine systematic changes over time in event amplitude, or open- and closed-dwell time distributions. This allowed us to ensure that effects measured were not influenced by a receptor run-down phenomenon as previously described by us and others for α4β2-nAChR [25,38]. For HS-isoform α4β2-nAChR containing either of the SHE-associated α4(R336H) or β2(V337G) mutant subunits, data were only analyzed during the first 60s of recordings. After this cut-off, stability plots indicated that functional properties for this isoform changed as a function of time. Function within patches containing LS-isoform α4β2-nAChR harboring either of these SHE-associated subunits was shown by the same stability plot approach to be more stable, allowing data from the first 120s to be analyzed.

Also as previously performed for wildtype HS- and LS-α4β2-nAChR isoforms [25], all individual patches corresponding to each studied combination of receptor composition and ACh concentration were examined for consistency in the number of events per second. A two standard deviations (SD) outlier test was applied to exclude from analyses those patches having event frequencies substantially smaller or larger than the mean. The rationale was that patches exhibiting unusually high numbers of events likely contained atypically large numbers of nAChR, whereas those patches displaying very few openings likely contained abnormally few and/or desensitized, inactivated, or run-down nAChR. After application of this further quality-control criterion, each experimental group contained 5–8 single-channel recordings that were used for further data analysis.

### Single-channel burst analysis

As previously observed for wildtype α4β2-nAChR isoforms [25], single-channel responses of the equivalent isoforms harboring SHE-associated α4(R336H) or β2(V337G) mutant subunits occurred as a mixture of isolated single openings, and less-frequent short bursts of channel opening, interspersed with longer-duration closed-dwell periods. Bursts were defined as series of two or more openings separated by closures shorter than a minimum interburst closed duration (or T_crit_) chosen to minimize the number of misclassified closed events [39,40]. Very few observed bursts contained multiple open amplitudes. When such behavior was observed, these bursts were discarded from analysis since they likely indicate that multiple channels were active simultaneously, and the intended outcome of burst analysis is to study multiple adjacent openings arising from a single receptor [41,42]. Burst properties, such as the proportion of openings found within bursts, numbers of openings within a burst, and open probability within a burst (P_open_) were quantified using QuB. Exported QuB data were used to determine burst duration values using exponential log probability histograms generated by Clampfit 10.4.1.4 software (Molecular Devices, San Jose, CA, USA).

Only a single open amplitude was seen for bursts arising from HS-isoform α4β2-nAChR containing SHE-associated α4(R336H) or β2(V337G) mutant subunits. However, bursts of activity from LS-isoform α4β2-nAChR containing either mutant subunit always fell into two populations, composed of open events with either small or large amplitudes. This production of two different populations of bursts matched that seen for wildtype LS-isoform α4β2-nAChR [25]. Accordingly, we applied the same procedure as in our previous publication: differentiation between bursts containing small or large amplitude openings was accomplished using the QuB X-means algorithm to separate the two populations [25,43], which were then analyzed separately.

### Comparisons between SHE-associated mutant to wildtype receptors

To avoid the need to duplicate here the presentation of already-published data, and to facilitate comparison of properties measured from α4β2-nAChR isoforms containing either of the two SHE-associated mutant subunits to those previously published for wildtype α4β2-nAChR, we present such comparisons in terms of “fold change” for each property. To accomplish this, the value for each property (determined for each patch exhibiting functional responses of α4β2-nAChR containing SHE-associated mutant subunits) was divided by the published [25] mean value of the corresponding property determined for the corresponding wildtype α4β2-nAChR isoform. This comparison is valid since recordings of wildtype α4β2-nAChR that were the subject of the previous publication, and those containing SHE-associated α4(R336H) or β2(V337G) mutant subunits that are subjects of this study, were performed in parallel, using the same conditions and reagents, and applying identical approaches to data analysis. The resulting fold-change values in each case were averaged across all comparable patches, to yield a mean ± SEM fold-change value for each recorded property. Values > 1 represent an increase in the analyzed property due to the introduction of an SHE-associated mutant subunit in comparison to wildtype receptors. Conversely, values < 1 represent a reduction of the property in the presence of an SHE-associated mutant subunit, when compared to the corresponding value calculated from wildtype receptors.

### Molecular visualization of SHE-associated mutant positions

To illustrate the structural locations of the intracellular SHE-associated mutations, the 5HT_3A_R closed (apo; PDB ID: 6BE1) and open (conducting; PDB ID: 6BE1) cryo-EM structures were used to build models using ChimeraX (version 0.93; University of California CA). Specifically, three residues of interest were substituted from 5HT_3A_ amino acids to the wildtype or SHE-associated α4 or β2 nAChR subunit equivalents. The spatial location of each amino acid sidechain that was swapped was selected using the Dunbrack rotamer library. For each substitution, the position of the residue with the highest probability of existing, the smallest number (if any) clashes, and no unexpected H-bonds were used in the generated models.

### Statistical analysis

Results are presented as mean ± standard error of the mean (S.E.M.), except for error estimates associated with Gaussian or exponential distributions (which are described as histograms with the best fit value ± S.E.M.). Prism 5.03 Software (La Jolla, CA, USA) was used to statistically analyze the measured single-channel functional properties. The number of patches included in each analysis is reported as N. Two-tailed Unpaired Student’s T-tests were used to compare pairs of groups. One-way or two-way analysis of variance (ANOVA) and Tukey’s multiple comparison tests were used to evaluate the means of three or more groups and differences between them. Fold change analysis was performed using a one-sample t test and comparison to a hypothetical value of 1.0, where 1.0 indicates no change.

## Results

For ease of identification, all data collected from α4β2-nAChR containing α4(R336H) subunits are presented throughout the Figures in green. Those collected from α4β2-nAChR containing β2(V337G) mutant subunits are shown in magenta. All experiments were performed contemporaneously with those of our recently-published study [25]. Experimental conditions, reagents, and analysis approaches were identical to those applied in that study. These features allow for valid comparisons to be made between observations of single channel properties of HS- or LS-α4β2-nAChR isoforms containing wildtype subunits (previous study), and those containing SHE-associated mutant subunits (current study). Changes resulting from the incorporation of a SHE-associated mutant subunit are expressed as fold-change value for each recorded property, as detailed in the *Experimental Procedures* section. Values over one represent an increase in the analyzed property due to the introduction of an SHE-associated mutant subunit in comparison to wildtype receptors. Conversely, values less than one represent a reduction of the property in the presence of an SHE-associated mutant subunit, when compared to the corresponding value calculated from wildtype receptors. This approach avoids the necessity of presenting again results that were already shown in our earlier publication, and focuses attention on changes induced by introduction of the mutant subunits (determination of which is a major objective of the current study).

### Incorporation of α4(R336H) subunits has minimal effects on open-channel amplitudes of HS- or LS-isoform α4β2-nAChR, whereas β2(V337G) subunit incorporation significantly reduces amplitudes of openings of both isoforms, when compared to their counterparts containing wildtype subunits

**Fig 1:**
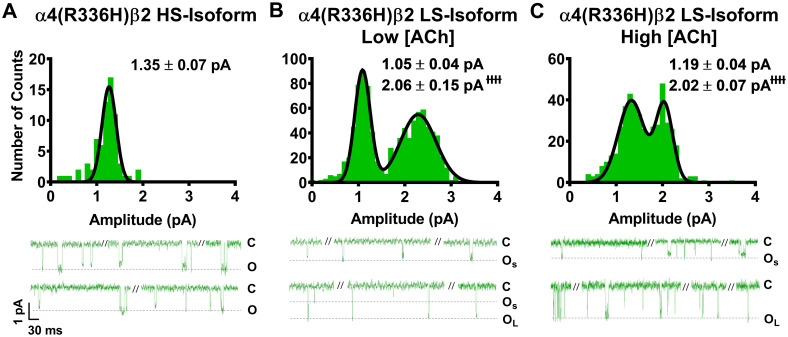
Unitary amplitudes associated with human HS- and LS-isoform α4(R336H)β2-nAChR expressed in *Xenopus laevis* oocytes. Example single-channel ACh evoked response traces are shown below panels (A), (B), and (C), exhibiting a typical mixture of individual openings and short bursts of activity, interspersed with longer periods of inactivity. (A) Amplitudes of HS-isoform α4(R336H)β2-nAChR open events evoked by 1.3 μM ACh are characterized as a single population. (B and C) LS-isoform α4(R336H)β2-nAChR single-channel openings appeared to exhibit two distinct amplitudes, whether they were evoked in the presence of either a low ACh concentration (0.7 μM; Panel B) or a high ACh concentration (30 μM; Panel C). This finding was confirmed by two-way ANOVA, using ACh concentration and apparent amplitude class (small opening (O_S_) or large opening (O_L_)) as factors. A main effect of amplitude class was observed, confirming that amplitudes of O_S_ and O_L_ are distinctly different from each other (F_1,22_ = 144.7, ^ɫɫɫɫ^P < 0.0001). In contrast, no main effect of ACh concentration on open amplitude was observed (F_1,22_ = 0.04, P = 0.53), nor was there a significant interaction between the two factors (interaction ACh concentration x amplitude size F_1,22_ = 0.25, P = 0.25). Accordingly, the amplitudes of O_S_ and large opening O_L_ were not significantly altered by changes in the concentration of ACh applied. Amplitude histograms represent events collected across multiple individual single-channel patch recordings, for each illustrated combination of receptor construct and ACh concentration. Values are given as mean ± S.E.M., and were collected from 5–8 patches across a minimum of three separate experiments.

**Fig 2:**
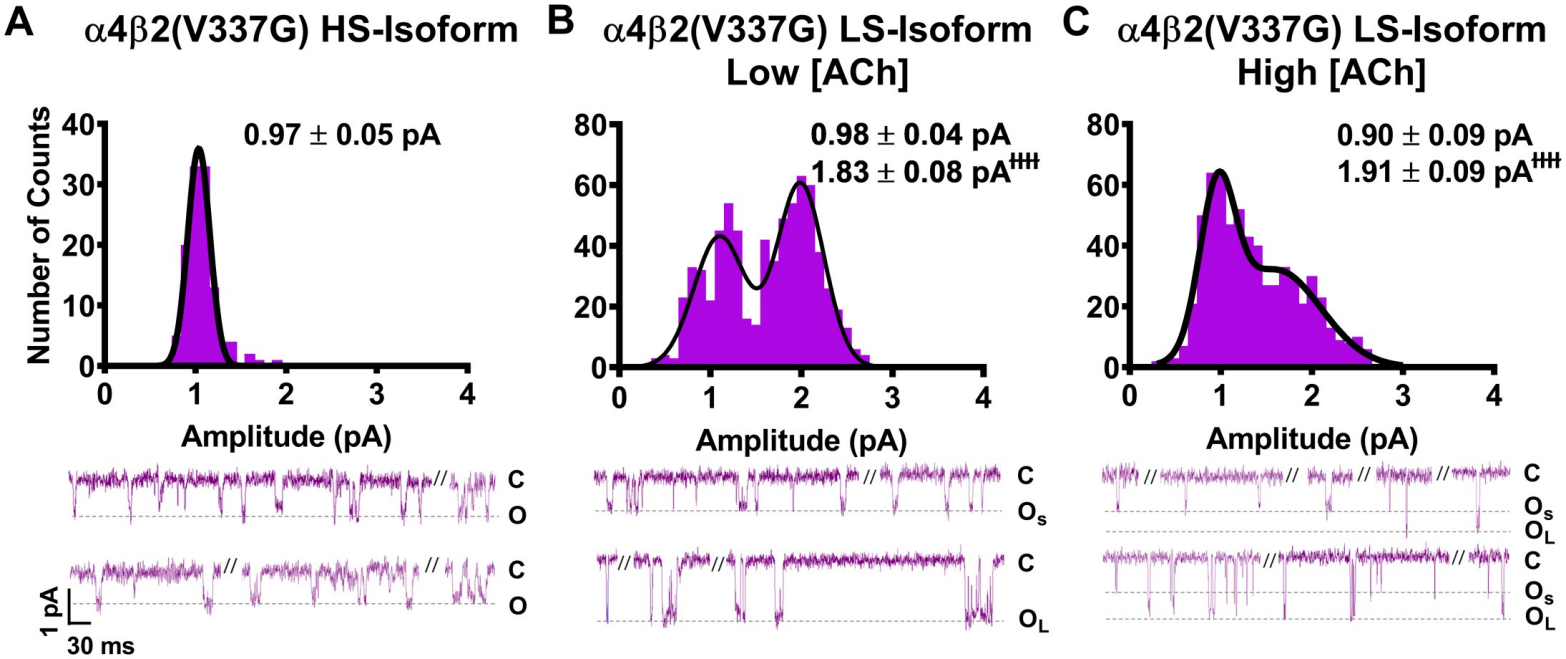
Unitary amplitudes associated with human HS- and LS-isoform α4β2(V337G)-nAChR expressed in *Xenopus laevis* oocytes. As in Fig 1, example single-channel ACh evoked response traces are shown below panels (A), (B), and (C), which display a mixture of individual openings and short bursts of activity, which are interspersed with longer periods of inactivity. (A) HS-isoform α4β2(V337G)-nAChR stimulated with 1.3 μM ACh produced single-channel openings with a single characteristic amplitude. (A) Amplitudes of HS-isoform α4(R336H)β2-nAChR open events evoked by 1.3 μM ACh are characterized as a single population. (B and C) LS-isoform α4β2(V337G)-nAChR single-channel openings appeared to exhibit two distinct amplitudes, whether they were evoked in the presence of either a low ACh concentration (0.7 μM; Panel B) or a high ACh concentration (30 μM; Panel C). Similar to the findings for LS-isoform α4(R336H)β2-nAChR, this was confirmed by two-way ANOVA. A main effect of amplitude class was again observed, confirming that amplitudes of small openings (O_S_) and large openings (O_L_) are distinctly different from each other (*F*_1,20_ = 124.2, ^ɫɫɫɫ^*P* < 0.0001). In a further point of similarity with LS-isoform α4(R336H)β2-nAChR, the amplitudes of O_S_ and O_L_ of LS-isoform α4β2(V337G)-nAChR were not significantly altered by changes of applied ACh concentration (F_1,22_ = 0.04, P = 0.53), nor was there a significant interaction between the two factors (interaction ACh concentration x amplitude size F_1,22_ = 0.25, P = 0.25). Amplitude histograms represent events collected across multiple individual single-channel patch recordings, for each illustrated combination of receptor construct and ACh concentration. Values are given as mean ± S.E.M, and were collected from 5–7 patches across a minimum of three separate experiments.

As noted in the introduction, our prior study [14] suggested that the primary macroscopic effects of incorporation of the two SHE-associated mutant subunits α4(R336H) and β2(V337G) are a gain-of-function-per-receptor and a bias towards HS-phase function in mixed populations of α4β2-nAChR HS- and LS-isoforms. To investigate the mechanism(s) by which these effects may arise, we first analyzed the amplitudes of unitary events induced by ACh. In this case, all events recorded (both individual openings, and openings within a burst) were analyzed using a transmembrane potential of -100 mV. As previously observed for wildtype α4β2-nAChR isoforms [25], HS-isoform α4β2-nAChR containing either mutant subunit revealed events that were of a single amplitude (Figs 1A and 2A). By contrast, LS-isoform α4β2-nAChR harboring either mutant subunit exhibited events with two distinct unitary amplitudes (O_S_ and O_L_ for openings with the smaller or the larger unitary amplitudes, respectively; Figs 1B, 1C, 2B and 2C).

Looking first at HS-isoform α4(R336H)β2-nAChR, function was stimulated with 1.3 μM ACh (corresponding to the EC_50_ value of the single phase of agonist response when measured macroscopically as whole-cell currents of this isoform). The amplitudes of individual openings (Fig 1A) were measured as 1.35 ± 0.07 pA. This was not significantly different from the amplitude previously recorded from wildtype HS-isoform α4β2-nAChR (Table 1). The openings of α4(R336H) LS-isoform α4β2-nAChR were recorded at two different ACh concentrations, corresponding to the EC_50_ values for stimulation of HS- (Fig 1B; 0.7 μM) or LS-phase (Fig 1C; 30 μM) responses when measured macroscopically as whole-cell currents for this isoform. The small amplitude openings (O_S_) observed were statistically indistinguishable from each other when recorded in the presence of either 0.7 μM or 30 μM ACh (1.05 ± 0.04 pA or 1.19 ± 0.04 pA, respectively). The same was true for the large amplitude openings (O_L_; 2.06 ± 0.15 pA or 2.02 ± 0.07 pA, respectively). As summarized in Table 1, only one of these unitary amplitude properties was significantly different between wildtype LS-isoform α4β2-nAChR and those incorporating the α4(R336H) subunit; a small but statistically significant drop in O_S_ (to 87 ± 3% of the wildtype value) was observed, at the lower ACh concentration.

**Table 1:** Unitary amplitudes of human HS- and LS-isoform α4(R336H)β2-nAChR.

Isoform	Unitary Amplitude ± SEM (pA)		
	Small	Large	Number of patches
α4(R336H)β2 HS	1.35 ± 0.07	n/a	8
Fold Change ± SEM	0.90 ± 0.04	n/a	n/a
*Low [ACh] (0*.*7 μM)*			
α4(R336H)β2 LS	1.05 ± 0.04	2.06 ± 0.15	5
Fold Change ± SEM	0.87 ± 0.03*	0.93 ± 0.07	n/a
*High [ACh] (30 μM)*			
α4(R336H)β2 LS	1.19 ± 0.04	2.02 ± 0.07	8
Fold Change ± SEM	1.02 ± 0.03	1.05 ± 0.04	n/a

Human α4(R336H)β2-nAChR subunits were expressed in *Xenopus laevis* oocytes as pure populations of either [α4(R336H)β2]_2_β2 stoichiometry (HS-isoform) or [α4(R336H)β2]_2_α4(R336H) stoichiometry (LS-isoform). Amplitudes of individual channel openings of these populations were measured as described in the *Experimental Procedures*. For HS-isoform α4(R336H)β2-nAChR, macroscopic concentration-response curves are monophasic. Therefore, single-channel events were elicited using a single ACh concentration (1.3 μM; macroscopic EC_50_). For LS-isoform α4(R336H)β2-nAChR, macroscopic concentration-response curves are biphasic, thus single-channel events were stimulated at two different ACh concentrations (0.7 μM or 30 μM). These correspond to the macroscopic EC_50_ values of the two different phases of the LS-isoform ACh concentration-response curve, respectively. As shown in Fig 1, openings of HS-isoform α4(R336H)β2-nAChR fell into a single amplitude class, while those of the LS-isoform α4(R336H)β2-nAChR fell into two classes. All properties shown are mean ± SEM of values derived from 5–8 individual patches, as noted. The value of each property was compared to the mean value of its counterpart measured (contemporaneously and under identical conditions) from wildtype α4β2-nAChR populations, using a One Sample T-test. In each case, the fold difference relative to the corresponding property of the wildtype α4β2-nAChR counterpart is reported as Fold Change ± SEM (see *Experimental Procedures* for detail). * Signifies statistically significant reduction in the small unitary amplitude response of the LS-isoform α4(R336H)β2-nAChR evoked with the low ACh concentration (0.7 μM) compared to wildtype receptors (* P < 0.05, t = 3.71, df = 4).

We next examined single-channel events of α4β2-nAChR incorporating the β2(V337G) subunit. HS-isoform α4β2(V337G)-nAChR unitary amplitudes had a mean amplitude of only 0.97 ± 0.05 pA (Fig 2A). This was significantly smaller (65 ± 4% of the wildtype value) than the amplitude previously recorded from wildtype HS-isoform α4β2-nAChR (Table 2). A similar trend was observed for unitary amplitude properties recorded from LS-isoform α4β2(V337G)-nAChR. The values of both O_S_ and O_L_ determined in the presence of 0.7 μM ACh (Fig 2B), and O_S_ in the presence of 30 μM ACh (Fig 2C) were significantly lower than those measured for wildtype LS-isoform α4β2-nAChR under identical conditions (each reduced to approximately 80% of its wildtype comparator). Only the value of O_L_ determined at the higher ACh concentration was unaffected (outcomes summarized in Table 2). In a point of similarity to the observations made from α4(R336H)β2-nAChR (preceding paragraph), the LS-isoform α4β2(V337G)-nAChR small amplitude openings (O_S_) observed were statistically indistinguishable from each other when recorded in the presence of either 0.7 μM or 30 μM ACh (0.98 ± 0.04 pA or 0.90 ± 0.09 pA, respectively). The same was true for the large amplitude openings (O_L_) (1.83 ± 0.08 pA or 1.91 ± 0.09 pA, respectively).

**Table 2:** Unitary amplitudes of human HS- and LS-isoform α4β2(V337G)-nAChR.

Isoform	Unitary Amplitude ± SEM (pA)		
	Small	Large	Number of patches
α4β2(V337G) HS	0.97 ± 0.05	n/a	6
Fold Change ± SEM	0.65 ± 0.04***	n/a	n/a
*Low [ACh] (0*.*7 μM)*			
α4β2(V337G) LS	0.98 ± 0.04	1.83 ± 0.08	5
Fold Change ± SEM	0.81 ± 0.03**	0.81 ± 0.04**	n/a
*High [ACh] (30 μM)*			
α4β2(V337G) LS	0.90 ± 0.09	1.91 ± 0.09	7
Fold Change ± SEM	0.77 ± 0.08*	0.99 ± 0.05	n/a

Using an approach identical to that described in Table 1 for α4(R336H)β2-nAChR, the amplitudes of individual openings of HS- or LS-isoform α4β2(V337G)-nAChR were determined, and compared to their counterparts measured from wildtype α4β2-nAChR isoforms using a One Sample T-test. As for Table 1 the fold difference, in each case, relative to the corresponding property of the wildtype α4β2-nAChR counterpart is reported as Fold Change ± SEM (see *Experimental Procedures* for detail). As for α4(R336H)β2-nAChR, openings of HS-isoform α4β2(V337G)-nAChR fell into a single amplitude class, while those of LS-isoform α4β2(V337G)-nAChR fell into two classes (see Fig 2). All properties shown are mean ± SEM of values derived from 5–7 individual patches (number of patches, N in each case is shown in the table). Incorporation of the SHE-associated β2(V337G) mutant subunit significantly reduced unitary amplitudes vs. their equivalents recorded from wildtype α4β2-nAChR isoforms in multiple cases: HS-isoform unitary amplitude (*** P < 0.001, t = 9.88, df = 5), both amplitude classes recorded from LS-isoform when stimulated with the low (0.7 μM) ACh concentration (small amplitude class ** P < 0.01, t = 5.70, df = 4; large amplitude class ** P < 0.01, t = 4.64, df = 4), and when the LS-isoform was stimulated at the high ACh concentration (30 μM), small amplitude openings were also significantly reduced in amplitude (* P < 0.05, t = 3.006, df = 6).

Our previous study clearly shows that incorporation these SHE-associated mutant subunits enhances macroscopic function-per-receptor [14]. However, the results of the current work demonstrate that the cytoplasmic loop SHE mutations minimally alter, in the case of the α4(R336H) mutation, or reduce, as seen with the β2(V337G) mutation, α4β2 nAChR unitary amplitudes. In neither case, therefore, can changes in unitary-event amplitudes induced by incorporation of α4(R336H) or β2(V337G) subunits be responsible for their previously-observed enhancement of macroscopic function-per-receptor.

### SHE-associated cytoplasmic loop mutations have differing effects on closed-dwell time durations between openings of α4β2-nAChR

We next examined closed-dwell time distributions for all events (including those between single events, and those within and between bursts). The briefest closed-dwell time component (τ_1_) in each case was considered to correspond to closed-dwell times within bursts, while longer components corresponded to closed events that occurred between individual openings, bursts, or both [44].

**Fig 3:**
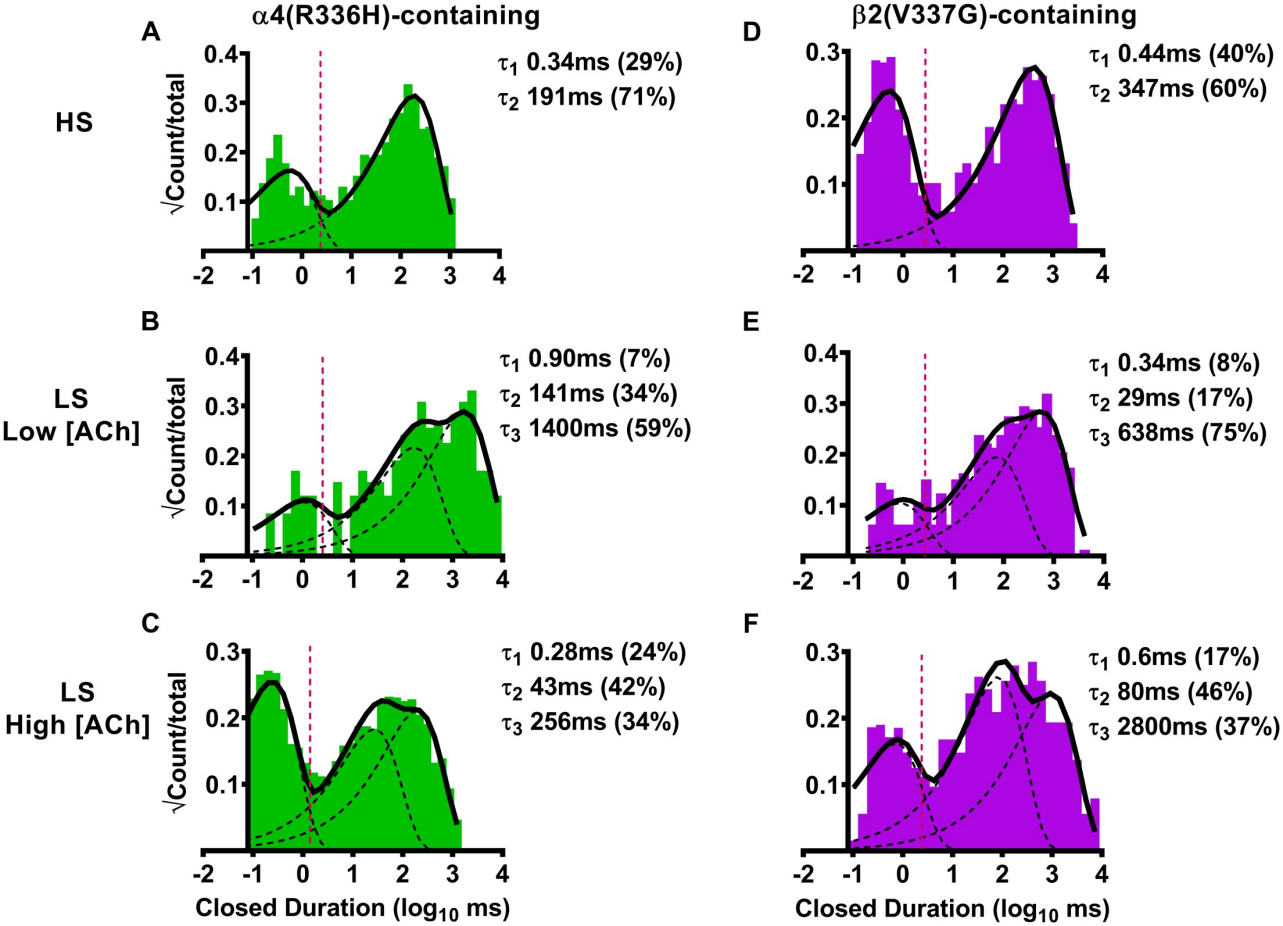
Closed-dwell time durations between single-channel openings of human HS- or LS-isoform α4(R336H)β2- or α4β2(V337G)-nAChR isoforms expressed in *Xenopus laevis* oocytes. (A) HS-isoform α4(R336H)β2-nAChR were stimulated with ACh (1.3 μM) and the resulting closed-dwell time durations between openings were best described with a pair of time constants. (B) When LS-isoform α4(R336H)β2-nAChR were stimulated at a low ACh concentration (0.7 μM), closed durations between openings were best described with three time constants. (C) The number of time constants required to best fit closed time distributions did not change as LS-isoform α4(R336H)β2-nAChR were stimulated at a higher ACh concentration (30 μM). (D) HS-isoform α4β2(V337G)-nAChR were stimulated with ACh (1.3 μM) and the resulting closed durations between openings were best described with a pair of time constants. (E) When LS-isoform α4β2(V337G)-nAChR were stimulated with a low ACh concentration (0.7 μM), closed durations between openings were best described with three time constants. (F) Increasing the ACh concentration to 30 μM did not change the number of time constants required to best fit closed duration distributions of LS-isoform α4β2(V337G)-nAChR. Closed-dwell duration histograms are representative examples collected from individual single-channel patch recordings. Individual τ values and percentage of total events corresponding to each closed duration (in parentheses) from these example patch recordings have been inserted into each panel to facilitate interpretation. Data were collected from 5–8 individual patches, across at least three separate experiments. Mean values of each property calculated from group data are summarized in Table 3, as mean ± SEM, together with any statistical analyses applied.

As for the preceding analysis of single-channel amplitudes, we began by examining effects of α4(R336H) subunit incorporation into HS-isoform α4β2-nAChR. In this case, the closed-dwell duration histogram was best fit with two closed durations, with most closed times falling into the longer-lived τ_2_ category (Fig 3A). As noted in Table 3, the number of closed durations, and the distribution of events found in the τ_2_ state did not differ significantly from what was previously observed for wildtype HS-isoform α4β2-nAChR. The number of events that fell into the τ_1_ state was significantly reduced (to 70 ± 10% in comparison to wildtype HS-isoform α4β2-nAChR). However, while within-burst closed times (τ_1_) were indistinguishable from those recorded from wildtype HS-isoform α4β2-nAChR, inclusion of the α4(R336H) subunit shortened the longer closed times (τ_2_) by half (summarized in Table 3). Considering next the effects of α4(R336H) subunit incorporation into LS-isoform α4β2-nAChR, the closed-dwell duration histogram in this case was best fit with three closed durations, at either ACh concentration Fig 3B and 3C, respectively and Table 3). Also, as summarized in Table 3, this number of closed durations, and the distribution of closed events across them, was statistically indistinguishable from that seen for wildtype LS-isoform α4β2-nAChR when stimulated at the same concentrations. Intriguingly, the durations of within-burst closed times (τ_1_) were consistently, and significantly, reduced for LS-isoform α4(R336H)β2-nAChR when stimulated with either ACh concentration (when compared to durations measured from their wildtype counterparts). Effects of α4(R336H) subunit incorporation on the remaining closed times were mixed, with no significant differences from wildtype values noted for τ_2_ at either ACh concentration applied, and a reduced value of τ_3_ seen at only the higher ACh concentration (Table 3).

**Table 3:** Closed-dwell time durations for human HS- and LS-isoform α4(R336H)β2- and α4β2(V337G)-nAChR.

Isoform	T_crit_ ± SEM	Closed Duration ± SEM (ms) (% ± SEM)		
		τ_1_	τ_2_	τ_3_
**α4(R336H)β2-nAChR**				
α4(R336H)β2 HS	3.6 ± 0.6	0.8 ± 0.2 (30 ± 4%)	250 ± 70 (70 ± 10%)	Absent
Fold Change ± SEM	--	0.9 ± 0.2 (0.7 ± 0.1*)	0.5 ± 0.1** (1.0 ± 0.2)	n/a
*Low [ACh] (0*.*7 μM)*				
α4(R336H)β2 LS	2.6 ± 0.7	0.6 ± 0.1 (14 ± 3%)	170 ± 40 (40 ± 10%)	2000 ± 800 (40 ± 10%)
Fold Change ± SEM	--	0.46 ± 0.09* (1.3 ± 0.2)	1.4 ± 0.3 (1.1 ± 0.3)	3 ± 1 (1.0 ± 0.3)
*High [ACh] (30 μM)*				
α4(R336H)β2 LS	1.4 ± 0.3	0.51 ± 0.04 (25 ± 6%)	19 ± 5 (38 ± 3%)	150 ± 50 (41 ± 5%)
Fold Change ± SEM	--	0.78 ± 0.06** (1.0 ± 0.2)	0.7 ± 0.2 (1.1 ± 0.1)	0.5 ± 0.2* (1.1 ± 0.1)
**β2(V337G)-nAChR**				
α4β2(V337G) HS	2.7 ± 0.4	1.0 ± 0.2 (27 ± 8%)	900 ± 400 (73 ± 8%)	Absent
Fold Change ± SEM	--	1.1 ± 0.3 (0.7 ± 0.2)	1.8 ± 0.7 (1.2 ± 0.1)	n/a
*Low [ACh] (0*.*7 μM)*				
α4β2(V337G) LS	2.5 ± 0.7	1.2 ± 0.5 (15 ± 5%)	60 ± 20 (30 ± 6%)	540 ± 60 (55 ± 9%)
Fold Change ± SEM	--	0.9 ± 0.4 (1.4 ± 0.5)	0.5 ± 0.1* (0.8 ± 0.1)	0.77 ± 0.08* (1.4 ± 0.2)
*High [ACh] (30 μM)*				
α4β2(V337G) LS	1.6 ± 0.4	0.6 ± 0.2 (12 ± 3%)	80 ± 20 (38 ± 7%)	2800 ± 800 (48 ± 9%)
Fold Change ± SEM	--	0.9 ± 0.3 (0.5 ± 0.1**)	3.0 ± 0.8 (1.1 ± 0.2)	10 ± 3* (1.3 ± 0.2)

HS- or LS-isoforms of α4(R336H)β2- or α4β2(V337G)-nAChR were expressed in *Xenopus laevis* oocytes as described in the *Experimental Procedures* section. Single-channel responses were evoked using either a single ACh concentration (for HS-isoform receptors; 1.3 μM) or at two different ACh concentrations (for LS-isoform receptors; 0.7 μM or 30 μM). Multiple closed-dwell times (τ_1_, τ_2_, *etc*.; see Fig 3) were determined between individual openings of these receptors and are shown as mean ± SEM of properties derived from 5–8 individual patches (exact numbers of patches in each case are given in Tables 1 and 2). In this analysis, closed-dwell times between all single-channel events were considered, whether or not they fell within bursts (although τ_1_ in each case corresponds to short closings within bursts of activity). Percentages of total events corresponding to each time constant are shown in parentheses under their associated time constants. The mean value of each property was compared to its counterpart measured (contemporaneously and under identical conditions) from wildtype α4β2-nAChR populations, using a One Sample T-test as detailed in the *Experimental Procedures* section. In each case, the fold difference relative to the corresponding property of the wildtype α4β2-nAChR counterpart is reported as Fold Change ± SEM.

For α4(R336H)β2-nAChR, HS-isoform closed-dwell time duration data were best fit with two dwell times, τ_1_ and τ_2_. Significant reductions were seen in the percentage of closed events falling within τ_1_ (* P < 0.05, t = 3.00, df = 7), and in the duration of the longer-lived τ_2_ closed events (** P < 0.006, t = 3.85, df = 7), when compared to values obtained from wildtype HS-isoform α4β2-nAChR. LS-isoform α4(R336H)β2-nAChR closed duration data were best fit with three closed-dwell time components, τ_1_, τ_2_, and τ_3_, regardless of the applied ACh concentration. Inclusion of the SHE-associated α4(R336H) subunit shortened the durations of several of the closed time classes when compared to those of wildtype LS-isoform α4β2-nAChR. At the low ACh concentration, the shortest closed-dwell time (τ_1_) was significantly reduced (* P < 0.05, t = 5.79, df = 3). At the higher ACh concentration both the longest and shortest closed-dwell times were significantly curtailed (τ_1_ ** P < 0.01, t = 3.75, df = 7; τ_3_ * P < 0.05, t = 2.64, df = 5).

For α4β2(V337G)-nAChR, HS-isoform closed duration data were again best fit with two components. Neither the durations of these two closed-dwell times, nor the proportions of events that fell into them, differed significantly from those recorded from wildtype HS-isoform α4β2-nAChR. The closed-dwell time distributions of LS-isoform α4β2(V337G)-nAChR were best described with three values, replicating the findings for wildtype LS-isoform α4β2-nAChR and α4(R336H)β2-nAChR. In a further point of similarity to α4(R336H)β2-nAChR, closed-dwell time durations of LS-isoform α4β2-nAChR were significantly reduced by incorporation of the β2(V337G) mutant subunit, in the presence of the low (0.7 μM) ACh concentration (τ_2_ * P < 0.05, t = 3.15, df = 4; τ_3_ * P < 0.05, t = 2.89, df = 4), in comparison to wildtype values. Increasing the ACh concentration to 30 μM produced a more-complex set of outcomes: the percentage of events falling into τ_1_ was significantly reduced (** P < 0.01, t = 5.00, df = 5), while the duration of events falling into τ_3_ was significantly lengthened (* P < 0.05, t = 3.37, df = 6), compared to values recorded from wildtype receptors.

We next examined closed-dwell properties of α4β2-nAChR incorporating the β2(V337G) subunit. The α4β2(V337G)-nAChR HS-isoform closed-dwell histogram was again best fit with two τ values (Fig 3D). However, in contrast to wildtype controls and to the HS-isoform α4(R336H)β2 mutant nAChR (just described), no significant changes in either the proportions of closed times associated with τ_1_ or τ_2_, or their durations were observed with the HS-isoform α4β2(V3367G) (Table 3). Moving next to the closed-dwell properties of LS-isoform α4β2-nAChR incorporating the β2(V337G) subunit, we observed that the LS-isoform closed time distribution was again best characterized using three closed-dwell time components for both applied ACh concentrations (Fig 3E and 3F). As shown in Table 3, the distributions of closed-dwell times among these three closed durations were mostly indistinguishable between these LS-isoform α4β2(V337G)-nAChR and their wildtype counterparts. The sole exception to this was observed at the high ACh concentration; the proportion of events that were associated with τ_1_ were about half of their wildtype counterpart values. However, several additional significant changes were noted in the durations of these closed-dwell times. When stimulated at the lower ACh concentration (0.7 μM, which evokes HS-phase responses from LS-isoform α4β2-nAChR), the longer closed-dwell times (τ_2_ and τ_3_) were significantly shortened for LS-isoform α4β2-nAChR containing the β2(V337G) subunit when compared to the corresponding values recorded for wildtype LS-isoform α4β2-nAChR. Conversely, at the higher ACh concentration (30 μM, which stimulates LS-phase activation of this isoform), the longest closed-dwell time between openings (τ_3_) was significantly extended in comparison to wildtype receptors. A similar trend was observed for τ_2_, but this did not quite reach significance (P = 0.055, t = 2.378, df = 6; One Sample T-test).

These results indicate that the two cytoplasmic SHE-associated mutations significantly alter the closed-dwell durations of α4β2-nAChR isoforms, albeit in distinctly different ways, and may contribute to the desensitization and/or inactivation kinetics that govern HS- and LS-isoform α4β2-nAChR function.

### The α4(R336H) SHE-associated mutation has no significant effects on HS- or LS-isoform α4β2-nAChR individual open-dwell times, whereas the β2(V337G) mutation prolongs a subset of open-dwell times for LS-isoform α4β2-nAChR

**Fig 4:**
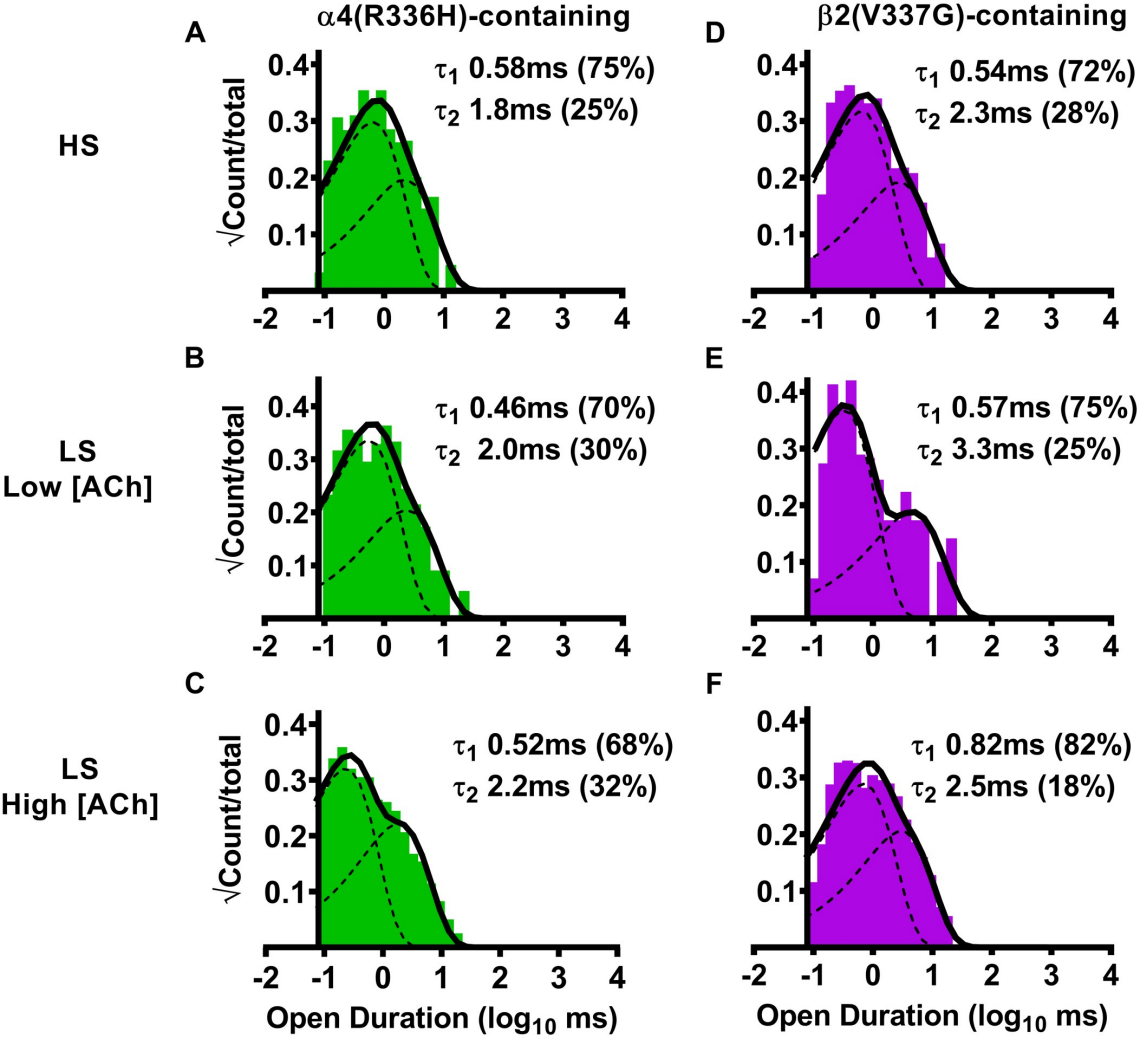
Open-dwell time durations observed for HS- or LS-isoform human α4(R336H)β2- and α4β2(V337G)-nAChR expressed in *Xenopus laevis* oocytes. (A) Stimulation of HS-isoform α4(R336H)β2-nAChR with ACh (1.3 μM) resulted in open- dwell-time durations best described with a pair of time constants. (B) Simulation of LS-isoform α4(R336H)β2-nAChR at a low ACh concentration (0.7 μM) also resulted in open durations that were best fit using two time constants. (C) The number of time constants required to best fit the open time distributions did not change as LS-isoform α4(R336H)β2-nAChR were stimulated at a higher ACh concentration (30 μM). (D) Simulation of HS-isoform α4β2(V337G)-nAChR with ACh (1.3 μM) resulted in open durations again best described with a pair of time constants. (E) When LS-isoform α4β2(V337G)-nAChR were stimulated with a low ACh concentration (0.7 μM), open-dwell times were also best fit using two time constants. (F) Increasing the ACh concentration to 30 μM did not change the number of time constants required to best fit the open duration distribution of LS-isoform α4β2(V337G)-nAChR. Open-dwell time histograms are representative examples resulting from analysis of individual single-channel patch recordings. Individual τ values and percentage of total events (in parentheses) corresponding to each open duration from these example patch recordings have been inserted into each panel to facilitate interpretation. Data were collected from 5–8 individual patches, across at least three separate experiments. Mean values of each property calculated from group data are summarized in Table 4, as mean ± SEM, together with any statistical analyses applied.

We next examined the duration of single-channel openings (all such openings, whether individual, or within bursts), since this represents another possible mechanism through which the SHE-associated mutant subunits could alter α4β2-nAChR function. We began by examining effects of α4(R336H) subunit incorporation into HS-isoform α4β2-nAChR. Open-dwell time histogram data in this case were best fit with two open-dwell time components, with the majority of events being short lived (Fig 4A, Table 4). Analyses revealed that no significant differences in HS-isoform α4(R336H)β2-nAChR open-dwell times, or the distributions of events, were observed compared to the wildtype HS-isoform α4β2-nAChR control (Table 4). A very similar outcome was observed for LS-isoform α4β2-nAChR hosting the α4(R336H) subunit. Open-dwell times were again best fit with two components (Fig 4B and 4C), with the majority of events falling into the shorter-lived category (Table 4). No statistically-significant differences were observed in any of these open-dwell times, or the distribution of those events when compared to those measured, in parallel and under identical conditions, from wildtype LS-isoform α4β2-nAChR.

**Table 4:** Open-dwell time duration properties for human HS- and LS-isoform α4(R336H)β2- and α4β2(V337G)-nAChR.

Isoform	Open Duration ± SEM (ms) (% ± SEM)	
	τ_1_	τ_2_
**α4(R336H)β2-nAChR**		
α4(R336H)β2 HS	0.6 ± 0.1 (64 ± 8%)	2.1 ± 0.4 (36 ± 6%)
Fold Change ± SEM	0.8 ± 0.2 (0.9 ± 0.1)	1.0 ± 0.2 (1.2 ± 0.3)
*Low [ACh] (0*.*7 μM)*		
α4(R336H)β2 LS	0.7 ± 0.1 (70 ± 10%)	2.3 ± 0.4 (30 ± 10%)
Fold Change ± SEM	1.4 ± 0.3 (0.9 ± 0.1)	1.0 ± 0.2 (1.4 ± 0.5)
*High [ACh] (30 μM)*		
α4(R336H)β2 LS	0.54 ± 0.10 (84 ± 4%)	1.8 ± 0.3 (22 ± 6%)
Fold Change ± SEM	1.5 ± 0.3 (1.2 ± 0.1)	1.3 ± 0.2 (0.7 ± 0.2)
**β2(V337G)-nAChR**		
α4β2(V337G) HS	0.55 ± 0.08 (70 ± 10%)	2.6 ± 0.2 (30 ± 10%)
Fold Change ± SEM	0.8 ± 0.1 (1.0 ± 0.2)	1.2 ± 0.1 (1.1 ± 0.4)
*Low [ACh] (0*.*7 μM)*		
α4β2(V337G) LS	0.57 ± 0.09 (75 ± 9%)	3.3 ± 0.4 (25 ± 9%)
Fold Change ± SEM	1.1 ± 0.2 (0.9 ± 0.1)	1.4 ± 0.2 (1.2 ± 0.5)
*High [ACh] (30 μM)*		
α4β2(V337G) LS	0.8 ± 0.2 (60 ± 10%)	2.3 ± 0.1 (40 ± 10%)
Fold Change ± SEM	2.1 ± 0.6 (0.9 ± 0.2)	1.66 ± 0.08**** (1.5 ± 0.5)

HS- or LS-isoforms of α4(R336H)β2- or α4β2(V337G)-nAChR were expressed in *Xenopus laevis* oocytes, and stimulated with ACh as described in the legend to Table 3. In all cases, open-dwell times of individual openings were best fit with two time constants (τ_1_ and τ_2_; see Fig 4). These properties are shown as mean ± SEM of values derived from 5–8 individual patches (exact numbers of patches are given in Tables 1 and 2). In this analysis, all openings were considered, whether or not they fell within bursts. Percentages of total events corresponding to each time constant are shown in parentheses under their associated time constants. The mean value of each property was compared to its counterpart measured (contemporaneously and under identical conditions) from wildtype α4β2-nAChR populations, using a One Sample T-test as detailed in the *Experimental Procedures* section. In each case, the fold difference relative to the corresponding property of the wildtype α4β2-nAChR counterpart is reported as Fold Change ± SEM.

For α4(R336H)β2-nAChR, all open duration data and the percentages of events associated with τ_1_ *vs*. τ_2_ were statistically indistinguishable from those collected from wildtype α4β2-nAChR under the same conditions. This was true for both the HS- and LS-isoforms when stimulated with either the lower or higher ACh concentration.

For α4β2(V337G)-nAChR, the same was true with only one exception. In this case, the longer open-dwell time (τ_2_) of LS-isoform α4β2(V337G)-nAChR was significantly extended in comparison that collected from its wildtype counterpart, but only when stimulated at the higher ACh concentration (τ_2_ **** P < 0.0001, t = 8.00, df = 7).

Next, we investigated the effects of β2(V337G) subunit incorporation, beginning with HS-isoform α4β2-nAChR. The open-dwell duration histogram of HS-isoform α4β2(V337G)-nAChR was also best fit with two open-dwell time components (Fig 4D). No statistically-significant differences were seen in either open-dwell time or in the relative proportion of short- *vs*. long-duration openings when compared to the wildtype HS-isoform α4β2-nAChR control (Table 4). A similar outcome was found when LS-isoform α4β2(V337G)-nAChR were stimulated with the lower (0.7 μM) ACh concentration (Fig 4E, Table 4); openings fell into two categories and the lengths of short- *vs*. long-duration openings and the distribution of openings between them remained indistinguishable from those of wildtype LS-isoform α4β2-nAChR. However, significant effects of including the β2(V337G) subunit were observed when the higher ACh concentration (30 μM) was applied (Fig 4F). While the proportion of short- *vs*. long-duration openings was statistically indistinguishable from that of wildtype LS-isoform α4β2-nAChR, fold-change analysis showed that the durations of the longer (τ_2_) state openings were significantly extended (Fig 4F, Table 4). This, therefore, represents the only significant observed effect of incorporation of either SHE mutant subunit in this initial, all events, analysis of open time properties, for either of the α4β2-nAChR HS- or LS-isoforms, in comparison to their wildtype receptor counterparts. However, as we will see in the following sections, a more-sophisticated analysis allows for single-channel events to be divided into distinct classes. This, in turn, uncovers important changes induced by incorporation of the α4(R336H) or β2(V337G) subunits.

### The α4(R336H) SHE-associated mutation decreases closed-dwell times between bursts of LS-isoform α4β2-nAChR openings, whereas the β2(V337G) mutation causes a variety of isoform-dependent changes

Our earlier work [25] demonstrated that LS-isoform α4β2-nAChR bursts are strictly segregated into two classes (those containing only small amplitude, or large amplitude, openings). Since this strongly suggests that LS-isoform α4β2-nAChR exhibit two distinct open states, we separated these two classes of bursts and analyzed them individually, as was done in our prior study (see *Experimental Procedures* section). This same approach was not performed for single-channel responses measured from HS-isoform α4β2-nAChR, since all openings of this isoform are of the same amplitude.

**Fig 5:**
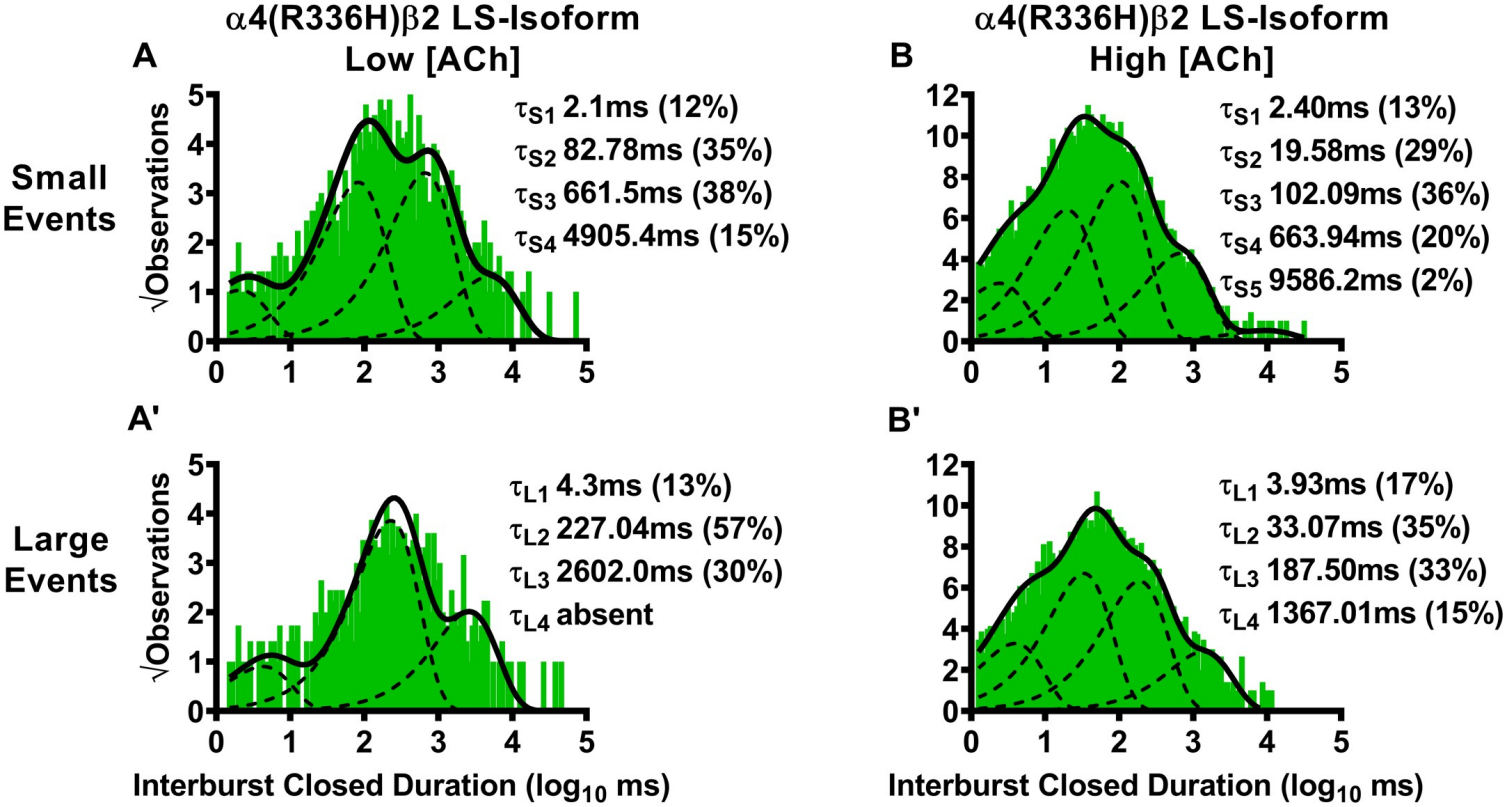
Closed-dwell time durations between burst activity of human LS-isoform α4(R336H)β2-nAChR. Closed-dwell time durations between bursts of activity (interburst durations) were measured for LS-isoform α4(R336H)β2-nAChR expressed in *Xenopus laevis* oocytes. Closed durations between bursts of either small (A) or large amplitude (A’) events evoked from LS-isoform α4(R336H)β2-nAChR at a low ACh concentration (0.7 μM) were best fit with four or three τ values, respectively. Increasing the ACh concentration to 30 μM resulted in LS-isoform α4(R336H)β2-nAChR closed state intervals between bursts of small (B) or large amplitude (B’) events that were best fit with five and four time constants, respectively. Interburst duration histograms are shown for pooled data from all recordings, which result from collection of data from 5 or 8 individual patches, across at least three separate experiments. The calculated τ values are summarized as mean ± SEM in Table 5, together with the statistical analyses applied.

We began by considering closed-dwell times between bursts (i.e., interburst intervals) of α4(R336H)β2 LS-isoform openings. First, this nAChR population was stimulated with a low (0.7 μM) concentration of ACh. For bursts of small amplitude openings (Fig 5A), the resulting interburst closed-dwell time histograms were best fit with four time components, with most events occurring in τ_S2_ and τ_S3_. For each of τ_S1_, τ_S2_, and τ_S3_, the interburst closed durations were significantly reduced compared to those previously measured for wildtype LS-isoform α4β2-nAChR (Table 5). Intriguingly, only one of the five patches analyzed exhibited bursts of large amplitude events, which precludes meaningful statistical analysis of the associated properties describing closed-dwell times between bursts of large amplitude openings. This reduction indicates a diminished prevalence of large amplitude bursts at low applied ACh concentrations when the α4(R336H) subunit is present. For the very few bursts of α4(R336H)β2 LS-isoform large amplitude events that were observed, the resulting interburst closed duration histogram was best fit with three closed-dwell time components (Fig 5A’). However, we caution that this analysis had to be performed on only the single patch that contained such events. Without corroboration across multiple patches, this finding may not be reliable.

**Table 5:** Closed-dwell time durations between bursts (interburst intervals) of human LS-isoform α4(R336H)β2- and α4β2(V337G)-nAChR.

Isoform	Interburst Closed Duration ± SEM (ms) (% ± SEM)								
	Small Amplitude					Large Amplitude			
	τ_S1_	τ_S2_	τ_S3_	τ_S4_	τ_S5_	τ_L1_	τ_L2_	τ_L3_	τ_L4_
**α4(R336H)β2-nAChR**									
*Low [ACh] (0*.*7 μM)*									
α4(R336H)β2 LS	2.1 ± 0.2 (12 ± 2%)	82.78 ± 0.08 (35 ± 2%)	661.5 ± 0.1 (38 ± 2%)	4905.4 ± 0.2 (15 ± 2%)	Absent	4.3 ± 0.3 (13 ± 2%)	227.04 ± 0.06 (57 ± 3%)	2602.0 ± 0.1 (30 ± 2%)	Absent
Fold Change ± SEM	0.17 ± 0.001**** (0.63 ± 0.03**)	0.64 ± 0.002**** (0.90 ± 0.08)	0.96 ± 0.01* (1.2 ± 0.1)	1.36 ± 0.01**** (1.7 ± 0.1**)	n/a	0.347 (0.5)	1.679 (1.5)	2.346 (1.0)	n/a
*High [ACh] (30 μM)*									
α4(R336H)β2 LS	2.40 ± 0.09 (13 ± 1%)	19.58 ± 0.07 (29 ± 1%)	102.09 ± 0.06 (36 ± 1%)	663.94 ± 0.07 (20 ± 1%)	9586.2 ± 0.4 (2 ± 1%)	3.93 ± 0.08 (17 ± 1%)	33.07 ± 0.06 (35 ± 1%)	187.50 ± 0.07 (33 ± 1%)	1367.01 ± 0.09 (15 ± 1%)
Fold Change ± SEM	0.543 ± 0.002**** (0.57 ± 0.02****)	0.51 ± 0.001**** (1.00 ± 0.04)	0.314 ± 0.001**** (1.00 ± 0.06)	0.351 ± 0.002**** (1.7 ± 0.1***)	Absent in WT	0.675 ± 0.002**** (0.71 ± 0.03****)	0.552 ± 0.001**** (1.03 ± 0.06)	0.372 ± 0.001**** (1.06 ± 0.06)	0.340 ± 0.002**** (1.25 ± 0.08*)
**β2(V337G)-nAChR**									
*Low [ACh] (0*.*7 μM)*									
α4β2(V337G) LS	26.00 ± 0.08 (33 ± 2%)	485.97 ± 0.09 (55 ± 4%)	2466.0 ± 0.4 (12 ± 4%)	Absent	Absent	2.3 ± 0.4 (12 ± 3%)	44.8 ± 0.2 (20 ± 3%)	395.6 ± 0.1 (41 ± 3%)	2798.3 ± 0.2 (28 ± 3%)
Fold Change ± SEM	2.097 ± 0.006**** (1.74 ± 0.09**)	3.75 ± 0.01**** (1.4 ± 0.2)	3.56 ± 0.06**** (0.38 ± 0.04***)	n/a	n/a	0.186 ± 0.002**** (0.44 ± 0.03****)	0.331 ± 0.002**** (0.51 ± 0.04***)	0.357 ± 0.001**** (1.3 ± 0.1*)	0.111 ± 0.003**** (9.3 ± 0.8***)
*High [ACh] (30 μM)*									
α4β2(V337G) LS	11.1 ± 0.1 (19 ± 2%)	154.20 ± 0.09 (35 ± 2%)	1378.4 ± 0.1 (33 ± 3%)	7659.7 ± 0.3 (13 ± 3%)	Absent	30.3 ± 0.1 (30 ± 2%)	560.2 ± 0.1 (40 ± 3%)	5949.6 ± 0.1 (30 ± 3%)	Absent
Fold Change ± SEM	2.511 ± 0.008**** (0.83 ± 0.05*)	4.02 ± 0.01**** (1.21 ± 0.07*)	4.29 ± 0.02**** (0.92 ± 0.09)	4.05 ± 0.04**** (1.1 ± 0.1)	n/a	5.21 ± 0.02**** (1.25 ± 0.07*)	9.36 ± 0.03**** (1.2 ± 0.1)	11.80 ± 0.04**** (0.97 ± 0.09)	n/a

**Fig 6:**
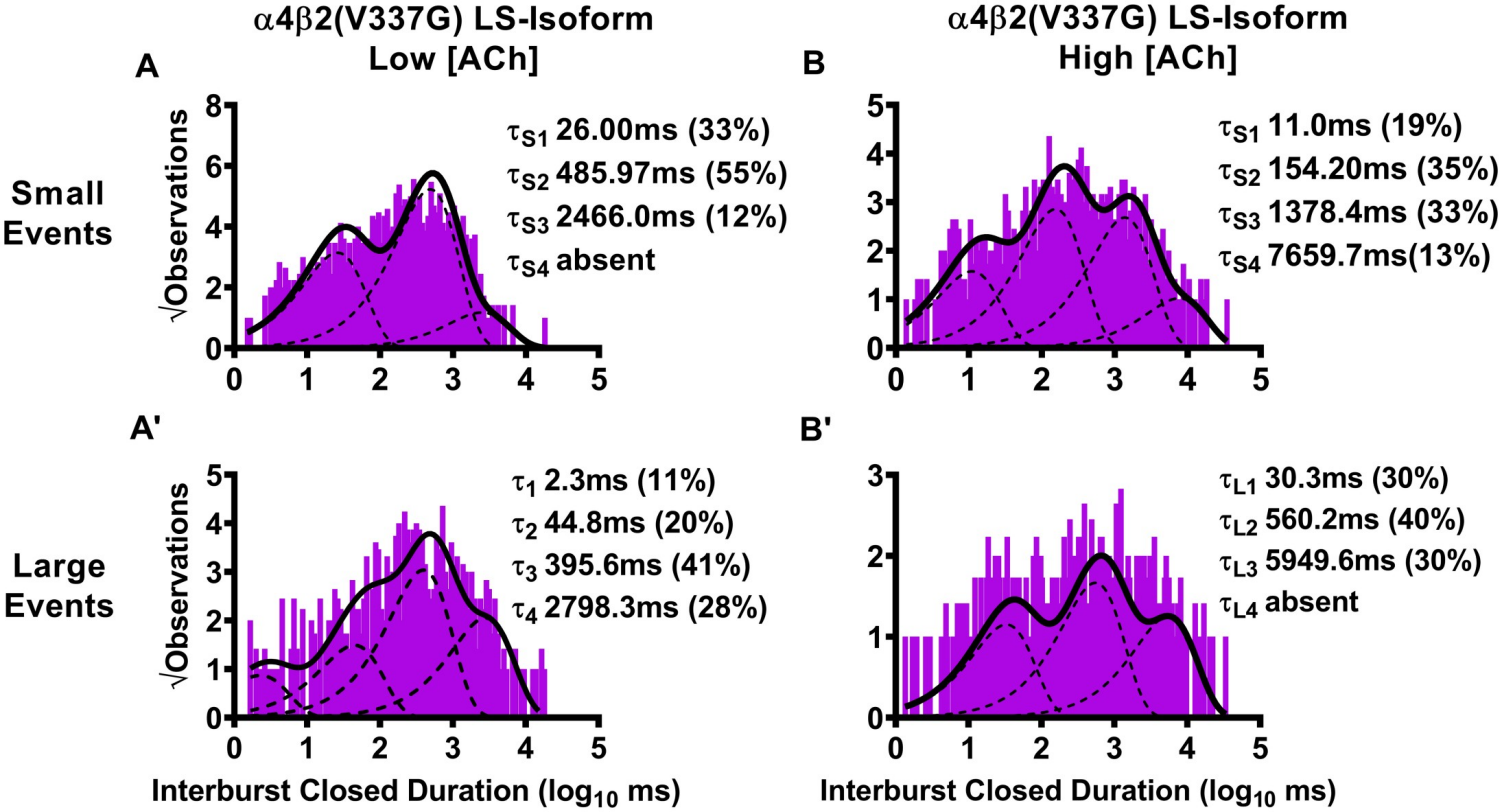
Closed-dwell time durations between burst activity of human LS-isoform α4β2(V337G)-nAChR. Closed-dwell time durations between bursts of activity (interburst closed durations) were measured for LS-isoform α4β2(V337G)-nAChR expressed in *Xenopus laevis* oocytes. Closed durations between bursts of either small (A) or large amplitude (A’) events evoked from LS-isoform α4β2(V337G)-nAChR at a low ACh concentration (0.7 μM) were best fit with either three or four τ values, respectively. Increasing the ACh concentration to 30 μM resulted in closed state intervals between bursts of LS-isoform α4β2(V337G)-nAChR small (B) or large amplitude (B’) events that were best fit with four and three time constants, respectively. Interburst closed duration histograms are shown for pooled data from all recordings, which result from collection of data from 5 or 7 individual patches, across at least three separate experiments. The calculated τ values are summarized as mean ± SEM in Table 5, together with the statistical analyses applied.

LS-isoform α4β2-nAChR exhibit bursts of either small or large amplitude openings. Accordingly, interburst intervals were assessed separately for bursts of each amplitude class of openings. Function was induced using two different ACh concentrations (0.7 μM or 30 μM). For each class of bursts, multiple interburst intervals (τ_1_, τ_2_, *etc*.) were observed, as illustrated in Fig 5 for α4(R336H)β2-nAChR, and Fig 6 for α4β2(V337G)-nAChR. The time constants associated with each interval are given in this table as mean ± SEM of properties measured from 5–8 individual patches, with the numbers of patches provided in Tables 1 and 2. Percentages of events associated with each time constant are shown in parentheses under their associated τ values. The mean value of each property was compared to its counterpart, determined in parallel and under identical conditions, from wildtype LS-isoform α4β2-nAChR, using a One Sample T-test as detailed in the *Experimental Procedures* section. In each case, the fold difference relative to the corresponding property of the wildtype α4β2-nAChR counterpart is reported as Fold Change ± SEM.

For LS-isoform α4(R336H)β2-nAChR stimulated with the low (0.7 μM) ACh concentration, interburst closed durations associated with small amplitude events were best fit with four closed-dwell time components. All of these were significantly shorter than their equivalents measured from wildtype LS-isoform α4β2-nAChR using the low (0.7 μM) ACh concentration (τ_S1_ **** P < 0.0001, t = 830.70, df = 4; τ_S2_ **** P < 0.0001, t = 181.00, df = 4; τ_S3_ * P < 0.05, t = 4.00, df = 4; τ_S4_ **** P < 0.0001, t = 35.58, df = 4). Together with this, a significant decrease in the percentage of events associated with the shortest interburst interval, accompanied by a significant increase in the proportion of events associated with the longest interburst interval, was observed (τ_S1_ ** P < 0.01, t = 6.17, df = 4; τ_S4_ ** P < 0.01, t = 7.00, df = 4). At the same low ACh concentration, LS-isoform α4(R336H)β2-nAChR large amplitude events were very rarely seen (and only in one patch out of five tested). This prevented statistical comparison of interburst closed durations to those measured for wildtype LS-isoform α4β2-nAChR. When observed, interburst intervals of LS-isoform α4(R336H)β2-nAChR under this condition were best fit with three closed-dwell time components.

For LS-isoform α4(R336H)β2-nAChR stimulated with the high (30 μM) ACh concentration, interburst intervals associated with bursts of small amplitude openings were best fit with five closed-dwell time components. This compares to only four components required to fit their wildtype receptor counterparts. All four of the shorter intervals were significantly curtailed compared to their wildtype receptor counterparts (τ_S1_ **** P < 0.0001, t = 228.50, df = 7; τ_S2_ **** P < 0.0001, t = 489.60 df = 7; τ_S3_ **** P < 0.0001, t = 686.00, df = 7; τ_S4_ **** P < 0.0001). Further, the percentage of events found within τ_S1_ was significantly reduced (**** P < 0.0001, t = 21.50, df = 7), accompanied by a significant increase in the proportion of events associated with the longest interburst interval τ_S4_ (*** P < 0.001, t = 7.00, df = 7). Intervals between bursts of large amplitude openings were best fit with four closed-dwell time components, the same as previously observed for wildtype LS-isoform α4β2-nAChR. Changes in interburst properties associated with large amplitude bursts induced by incorporation of the SHE-associated α4(R336H) mutant closely paralleled those seen for small amplitude bursts. All four of the interburst intervals were again significantly shorter than those measured from wildtype receptors τ_L1_ **** P < 0.0001, t = 162.40, df = 7; τ_L2_ **** P < 0.0001, t = 292.60, df = 7; τ_L3_ **** P < 0.0001; t = 628.10, df = 7; τ_L4_ **** P < 0.001, t = 330.00, df = 7). In addition, the proportion of events associated with the shortest interburst intervals was again significantly reduced τ_L1_ (**** P < 0.0001, t = 9.67, df = 7), and accompanied by a significant increase in the proportion of events associated with the longest interburst interval τ_S4_.

For LS-isoform α4β2(V337G)-nAChR stimulated with the low (0.7 μM) ACh concentration, interburst closed-dwell time durations associated with bursts of small amplitude openings were best fit with only three closed-dwell time components. All of these of were significantly *extended* compared to those measured from wildtype receptors τ_S1_ **** P < 0.0001, t = 185.90, df = 4; τ_S2_ **** P < 0.0001, t = 274.60, df = 4; τ_S3_ **** P < 0.0001, t = 42.69, df = 4). In addition, a significant increase in the percentage of the shortest interburst intervals τ_S1_ ** P < 0.01, t = 8.22, df = 4), accompanied by a reduction in that of the longest interburst intervals (τ_S3_ *** P < 0.01, t = 15.50, df = 4), was observed. Intervals between bursts of large amplitude openings were best fit with four closed-dwell time components, the same as required to describe those of wildtype LS-isoform α4β2-nAChR under the same conditions. Unlike the interburst intervals associated with small amplitude events, however, each of the intervals was significantly *shortened* compared to its wildtype counterpart (τ_L1_ **** P < 0.0001, t = 407.20, df = 4; τ_L2_ **** P < 0.0001, t = 255.30, df = 4; τ_L3_ **** P < 0.0001, t = 643.30, df = 4; τ_L4_ **** P < 0.0001, t = 296.20, df = 4). Also, in opposition to the findings for bursts of small amplitude events, the proportions of large amplitude events associated with the two shorter interburst intervals were reduced (τ_L1_ **** P < 0.0001, t = 18.67, df = 4; τ_L2_ *** P < 0.001, t = 12.25, df = 4), while the proportions associated with the longest interburst intervals were significantly increased (τ_L3_ * P < 0.05, t = 3.00, df = 4; τ_L4_ *** P < 0.001, t = 10.38, df = 4), compared to those observed from their wildtype receptor counterparts.

For LS-isoform α4β2(V337G)-nAChR stimulated with the high (30 μM) ACh concentration, durations between bursts of small amplitude openings were best classified into four populations. The time constants associated with all of these populations were elongated compared to their wildtype counterparts (τ_S1_ **** P < 0.0001, t = 188.90, df = 6; τ_S2_ **** P < 0.0001, t = 302.00, df = 6; τ_S3_ **** P < 0.0001, t = 164.40, df = 6; τ_S4_ **** P < 0.0001, t = 76.24, df = 6). The proportions of closed-dwell times associated with each interval were, in general, relatively unaffected by the presence of the β2(V337G) subunit, with a slight but significant increase in the proportion of the shortest intervals (τ_S1_ * P < 0.05, t = 3.40, df = 6), a minor but significant increase in the proportion of intervals associated with τ_S2_ (* P < 0.01, t = 3.00, df = 6), and no changes in the proportions of events associated with either τ_S3_ or τ_S4_. The outcomes for closed durations between bursts of large amplitude openings were best fit with three closed components, but the overall findings were otherwise similar to those for small amplitude bursts under this condition. All three interburst intervals were significantly extended (τ_L1_ **** P < 0.0001, t = 210.0, df = 6; τ_L2_ **** P < 0.0001, t = 278.50, df = 6; τ_L3_ **** P < 0.0001, t = 270.00, df = 6), and the proportions of events falling into each category remained generally similar (with the exception of a small but significant increase in the proportion of events falling into τ_L1_ (* P < 0.05, t = 3.0, df = 6).

We next examined closed-dwell times between bursts of α4(R336H)β2-nAChR LS-isoform openings, in the presence of the higher (30 μM) ACh concentration. For bursts of small amplitude openings (Fig 5B), the resulting interburst closed-dwell time histograms were best fit with five time components. This was itself a distinctly different outcome to that measured for wildtype LS-isoform α4β2-nAChR (which were best fit, in the presence of 30 μM ACh, with four time constants). Also of note, for each of τ_L1_, τ_L2_, τ_L3_, and τ_L4_, the interburst closed-dwell durations were significantly reduced compared to those previously measured for wildtype LS-isoform α4β2-nAChR under the same conditions (Table 5). A similar outcome was seen for closed-dwell times between bursts of large amplitude openings. Whereas only four time components were required to fit the closed-dwell time histogram in this case (the same as for wildtype LS-isoform α4β2-nAChR), every closed-dwell duration was shortened by incorporation of the α4(R336H) subunit (Table 5).

Moving to consider the effects of incorporating the β2(V337G) SHE-associated mutant subunit, we started by stimulating with the low ACh concentration (0.7 μM). Two striking changes were noted compared to the outcomes previously associated with small amplitude bursts under this condition for wildtype LS-isoform α4β2-nAChR. First, the interburst closed-dwell time histogram was best fit by only three properties (Fig 6A) as opposed to four for the wildtype equivalent. Second, those closed time properties were all significantly lengthened compared to those measured from analysis of the wildtype equivalent (Table 5). Intriguingly, the opposite effect was noted when closed-dwell times between bursts of large amplitude openings were examined (Fig 6A’). In this case, the interburst closed-dwell time histogram was best fit by four properties—but for each of τ_L1_, τ_L2_, τ_L3_, and τ_L4_, the interburst closed durations were significantly reduced relative to those for wildtype LS-isoform receptors (Table 5).

Last, we examined closed-dwell times between bursts of α4β2(V337G)-nAChR LS-isoform openings in the presence of the higher (30 μM) ACh concentration. In this context, all closed-dwell time properties measured, whether between bursts of small amplitude openings (Fig 6B), or between bursts of large amplitude openings (Fig 6B’), were significantly elongated compared to those measured under the same condition for wildtype LS-isoform α4β2-nAChR (Table 5). Effects of including the β2(V337G) mutant subunit also extended to the number of properties measured. Whereas the interburst closed-dwell time histogram between bursts of small amplitude events (Fig 6B) was best fit with four properties (as previously observed for wildtype LS-isoform α4β2-nAChR), the best fit between bursts of large amplitude openings was provided by only three properties (Fig 6B’).

In summary, these results showed that the α4(R336H) SHE mutation appears predominantly to decrease the time that LS-isoform α4β2-nAChR spend closed between bursts of openings. The only exception to this broad conclusion is that when the α4(R336H) mutation is incorporated into the LS-isoform, and this population is stimulated with the low ACh concentration, the longer-lived closed states between bursts of large amplitude events appeared to be lengthier than their wildtype counterparts. However, this observation comes with the caveat that bursts of large amplitude events were only seen in a single patch out of five recorded from under these conditions, making a reliable statistical comparison impossible. These interburst results reinforce the idea (originally noted when examining closed states between all openings, not just those associated with bursts), that the α4(R336H) mutation increases receptor function by decreasing the length of time the receptor stays closed between open events. The situation for the β2(V337G) SHE mutation is more complex, at least at the lower ACh concentration. In this LS-isoform, closed interburst interval events associated with bursts of small openings are longer than those measured for wildtype receptors, but those between bursts of large amplitude events are shortened. Application of the higher ACh concentration resulted in the intervals between bursts of both amplitude classes being significantly extended compared to those of their wildtype counterparts. This change would be expected to reduce macroscopic function-per-receptor, since it will result in each member of the receptor population, on average, spending more time in a closed state between openings. Accordingly, the macroscopic gain-of-function-per-receptor induced by incorporation of β2(V337G) subunits cannot be explained by this change in single-channel properties.

### Analysis of open-dwell times within LS-isoform bursts: The α4(R336H) SHE-associated mutation has mixed effects on durations of openings within bursts when compared to those for wildtype receptors, whereas the β2(V337G) mutation predominantly extends the durations of open events

We also analyzed the durations of the individual openings within bursts of LS-isoform α4β2-nAChR harboring either of the two SHE-associated mutant subunits. As for the preceding analysis of closed times between bursts, we measured these properties separately within bursts of small or large amplitude openings.

**Fig 7:**
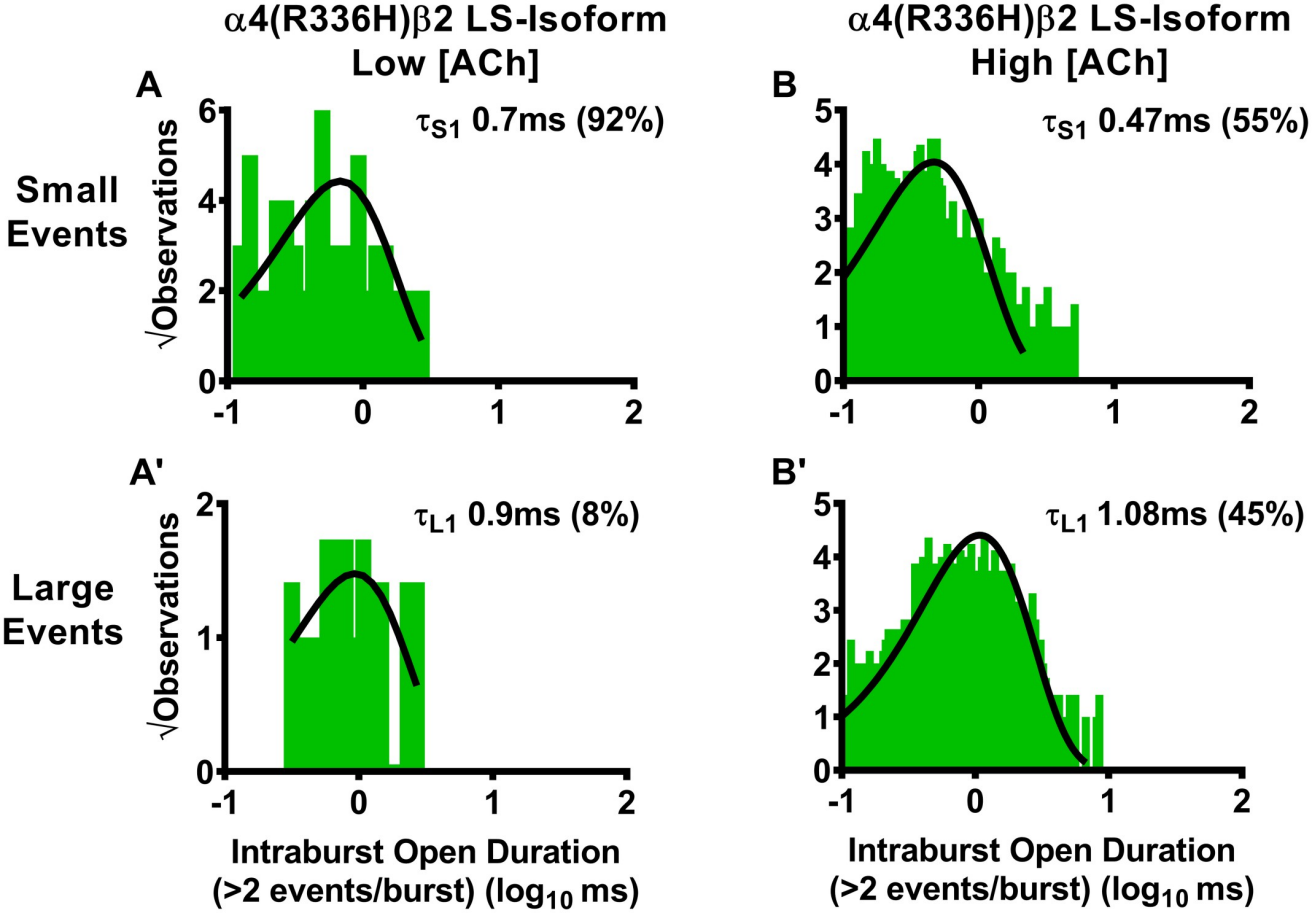
Durations of individual openings within bursts of small or large amplitude openings of human LS-isoform α4(R336H)β2-nAChR. LS-isoform α4(R336H)β2-nAChR were expressed in *Xenopus laevis* oocytes and durations of individual openings within bursts of activity (intraburst openings) were measured. For LS-isoform α4(R336H)β2-nAChR stimulated with a low ACh concentration (0.7 μM), bursts of either small (A) or large amplitude (A’) events were seen. Openings within bursts of small amplitude events were best characterized using a single time constant, as were openings within bursts of large amplitude events. When the ACh concentration was increased to 30 μM, durations of individual openings of LS-isoform α4(R336H)β2-nAChR within small (B) or large amplitude (B’) bursts each remained associated with single time constants. Histogram panels each show pooled data, which were collected from 5 or 8 individual patches, across at least three separate experiments. The calculated τ values are summarized as mean ± SEM in Table 6, together with the statistical analyses applied.

Beginning with the α4(R336H)β2-nAChR LS-isoform, we first considered the outcome of stimulating with the low ACh concentration (0.7 μM). Under this condition, both small and large amplitude openings within bursts were each best fit with a single time component (Fig 7A and 7A’, respectively; data summarized in Table 6). As noted in the preceding section, application of the low ACh concentration to LS-isoform α4(R336H)β2-nAChR evoked mostly events that fell within the small amplitude size category, with very few large amplitude events being observed (and these only within a single patch). The durations of the small amplitude openings elicited by 0.7 μM ACh were significantly shorter than those measured previously from wildtype LS-isoform α4β2-nAChR. No valid statistical comparison could be performed for the large amplitude openings within bursts under this low ACh concentration condition, since they were only observed in a single patch out of five recorded from. When the same α4(R336H)β2-nAChR LS-isoform population was stimulated with the higher ACh concentration, both small and large amplitude openings within bursts were again each best characterized with a single time constant (Fig 7B and 7B’, respectively). As noted in Table 6, at this higher ACh concentration, incorporation of the α4(R336H) subunit had mixed effects, significantly reducing the durations of small amplitude openings, but extending those of large amplitude openings, when compared to their equivalents recorded from wildtype α4β2-nAChR.

**Table 6:** Open-time properties within bursts (intraburst open duration) of human LS-isoform α4(R336H)β2- and α4β2(V337G)-nAChR.

Isoform	Individual Open Duration Within Bursts (Intraburst) ± SEM (ms) (% ± SEM)		
	Small Amplitude		Large Amplitude
	τ_S1_	τ_S2_	τ_L1_
**α4(R336H)β2-nAChR**			
*Low [ACh] (0*.*7 μM)*			
α4(R336H)β2 LS	0.7 ± 0.1 (92 ± 8%)	Absent	0.9 ± 0.2 (8 ± 8%)
Fold Change ± SEM	0.693 ± 0.002**** (1.8 ± 0.4)	n/a	0.542 (0.17)
*High [ACh] (30 μM)*			
α4(R336H)β2 LS	0.47 ± 0.05 (55 ± 9%)	Absent	1.08 ± 0.03 (45 ± 9%)
Fold Change ± SEM	0.839 ± 0.002**** (0.8 ± 0.3)	n/a	1.256 ± 0.002**** (1.3 ± 0.4)
**β2(V337G)-nAChR**			
*Low [ACh] (0*.*7 μM)*			
α4β2(V337G) LS	0.5 ± 0.2 (53 ± 8%)	4.0 ± 0.3 (47 ± 7%)	3.4 ± 0.1 (38 ± 9%)
	(62 ± 9%)		
Fold Change ± SEM	0.495 ± 0.002**** (1.0 ± 0.3)	3.96 ± 0.03**** (0.9 ± 0.2)	2.048 ± 0.005**** (0.8 ± 0.2)
*High [ACh] (30 μM)*			
α4β2(V337G) LS	0.9 ± 0.1 (42 ± 16%)	Absent	1.9 ± 0.2 (58 ± 16%)
Fold Change ± SEM	1.61± 0.01**** (0.6 ± 0.3)	n/a	2.21 ± 0.01**** (1.7 ± 0.8)

**Fig 8:**
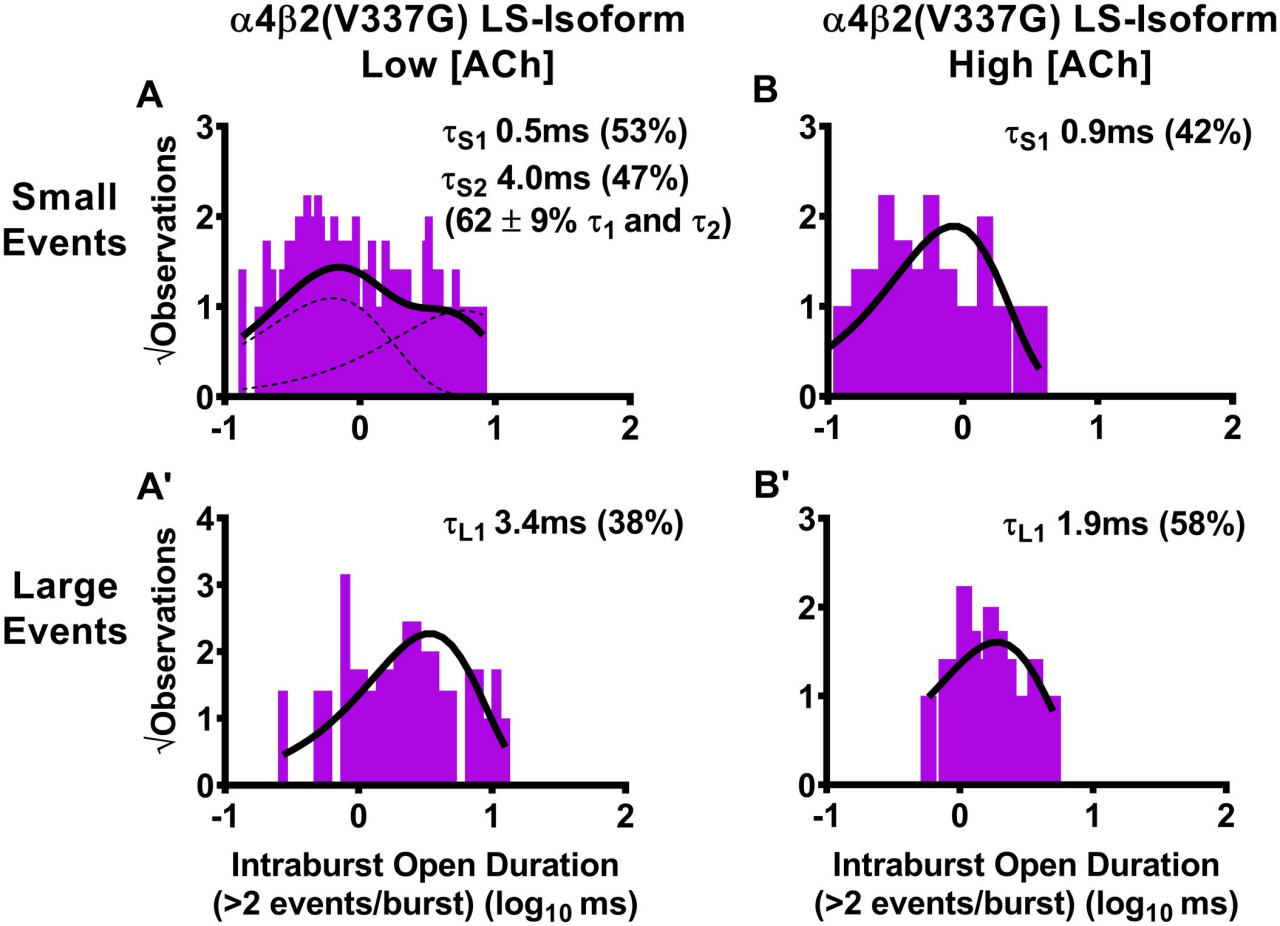
Durations of individual openings within bursts of small or large amplitude openings of human LS-isoform α4β2(V337G)-nAChR. LS-isoform α4β2(V337G)-nAChR were expressed in *Xenopus laevis* oocytes and durations of individual openings within bursts of activity (intraburst openings) were measured. When LS-isoform α4β2(V337G)-nAChR were stimulated with a low ACh concentration (0.7 μM), bursts of either small (A) or large (A’) amplitude events were seen. The open durations for individual events within bursts of small amplitude openings were best fit with two time constants (τ_1_ and τ_2_), while individual openings within bursts of large amplitude events were best fit with a single time constant. When the ACh concentration was increased to 30 μM, intraburst open durations of individual small amplitude openings of LS-isoform α4β2(V337G) nAChR within bursts (B) were best characterized using a single time constant. This contrasts with the two distinct time constants measured for small amplitude openings at the low ACh concentration. Individual open durations of large amplitude events within bursts remained best described with a single time constant, in the presence of the high ACh concentration (B’). Histogram panels each show pooled data, which were collected from 5 or 7 individual patches, across at least three separate experiments. The calculated τ values are summarized as mean ± SEM in Table 6, together with the statistical analyses applied.

Similar to the approach used in Table 5, open-times within bursts (intraburst open duration) of LS-isoform α4(R336H)β2- and α4β2(V337G)-nAChR were assessed separately for bursts of either small or large amplitude openings. Also as in the preceding analysis of closed-times between bursts, function was induced using two different ACh concentrations (0.7 μM or 30 μM). Intraburst open durations and distributions are depicted in Fig 7 for α4(R336H)β2-nAChR, and Fig 8 for α4β2(V337G)-nAChR. The time constants associated with intraburst openings in each case are given in this table as mean ± SEM of properties measured from 5–8 individual patches, with the numbers of patches provided in Tables 1 and 2. Percentages of events associated with each time constant are shown in parentheses under their associated τ values. The mean value of each property was compared to its counterpart, determined in parallel and under identical conditions, from wildtype LS-isoform α4β2-nAChR, using a One Sample T-test as detailed in the *Experimental Procedures* section. In each case, the fold difference relative to the corresponding property of the wildtype α4β2-nAChR counterpart is reported as Fold Change ± SEM.

For LS-isoform α4(R336H)β2-nAChR stimulated with the low (0.7 μM) ACh concentration, the open-dwell times of small amplitude openings within bursts were best fit with a single time constant that was significantly shorter than that associated with its wildtype counterpart (τ_S1_ **** P < 0.0001, t = 153.5, df = 4). At the same low ACh concentration, only one patch out of five tested contained any bursts of large amplitude openings. This precluded statistical comparisons of the properties obtained with those from wildtype LS-isoform α4β2-nAChR. The modest number of such openings observed was best fit with a single open-dwell time class. The proportion of openings falling into the large amplitude class appeared to be much lower than that associated with wildtype LS-isoform α4β2-nAChR, but statistical comparisons were not possible since, in the presence of 0.7 μM ACh, large amplitude bursts were only observed in a single patch.

For LS-isoform α4(R336H)β2-nAChR stimulated with the high (30 μM) ACh concentration, the open-dwell times of small amplitude openings within burst were again best fit with a single time constant. Similar to the situation when stimulated with the lower agonist concentration, these openings were again significantly shorter than that those of wildtype LS-isoform α4β2-nAChR when exposed to 30 μM ACh (τ_S1_ **** P < 0.0001, t = 80.36, df = 7). At the higher ACh concentration, bursts of large amplitude openings were consistently observed across all patches recorded from. These openings within bursts of large amplitude events were significantly longer than those produced by wildtype LS-isoform α4β2-nAChR (τ_L1_ **** P < 0.0001, t = 127.90, df = 7). The proportion of openings falling into either amplitude class at the high ACh concentration was statistically indistinguishable from that associated with wildtype LS-isoform α4β2-nAChR.

For LS-isoform α4β2(V337G)-nAChR, stimulated with the low (0.7 μM) ACh concentration, the open-dwell times of small amplitude openings within bursts were best fit with a pair of time constants, τ_S1_ and τ_S2_. These were, respectively, significantly shorter or longer (τ_S1_ **** P < 0.0001, t = 252.5, df = 4; τ_S2_ **** P < 0.0001, t = 98.67, df = 4), than the single time constant associated with openings of wildtype LS-isoform α4β2-nAChR that were stimulated at the same ACh concentration. Large amplitude openings evoked by the low ACh concentration fell into a single open-time distribution. The lengths of these openings were significantly extended compared to those of their wildtype receptor counterpart (τ_L1_ **** P < 0.0001, t = 209.6, df = 4). The proportion of small *vs*. large amplitude events evoked from LS-isoform α4β2(V337G)-nAChR was not different to that observed, in parallel, from wildtype LS-isoform α4β2-nAChR under the same conditions.

For LS-isoform α4β2(V337G)-nAChR, stimulated with the high (30 μM) ACh concentration, openings within small amplitude openings bursts were best described with a single time constant. This was significantly longer than that associated with openings of wildtype LS-isoform α4β2-nAChR under the same conditions (τ_S1_ **** P < 0.0001, t = 60.71, df = 6). Similarly, openings within bursts of large amplitude openings bursts were best described with a single time constant that was also significantly elongated compared to its wildtype counterpart (τ_L1_ **** P < 0.0001, t = 120.9, df = 6). No change in the proportion of events distributed between the small and large amplitude classes was noted at the higher ACh concentration.

Moving next to the function of the LS-isoform α4β2(V337G) population, we first analyzed single-channel events elicited with the low (0.7 μM) ACh concentration. Surprisingly, we found that openings within small amplitude bursts were best fit with two open-dwell time components, with an approximately similar distribution of events between the two components (τ_S1_ and τ_S2_; Fig 8A). As summarized in Table 6, the durations of these two open-dwell time components were approximately half as long (for τ_s1_), or four times longer (for τ_s2_), than that of the single open-dwell duration measured for wildtype receptors. At the same concentration of ACh, openings within large amplitude bursts were best fit with a single time component (Fig 8A’), and were approximately twice as long as those measured for large amplitude openings of wildtype LS-isoform α4β2-nAChR (Table 6). However, in the presence of the high ACh concentration (30 μM), the outcome was more straightforward. Only single time constants were needed to fit each of the open-dwell time distribution of openings within bursts of small or large amplitude openings (Fig 8B and 8B’, respectively). In both cases, the individual openings were significantly elongated compared to those measured for wildtype LS-isoform α4β2-nAChR under identical conditions (Table 6).

In summary, introduction of the α4(R336H) subunit has mixed effects on open times within bursts, but generally favors shorter openings that would be expected to reduce function-per-receptor. Accordingly, this change in single-channel behavior cannot be a basis for our previous observation of increased macroscopic function-per-receptor [14]. In contrast, incorporation of the β2(V337G) subunit generally results in prolongation of open times within bursts. This effect would be expected to result in increased function-per-receptor and, therefore, seems likely to contribute to the previously observed increase in macroscopic function-per-receptor [14].

### Burst analysis of LS-isoform α4β2-nAChR function: Proportion of bursts within all open events, numbers of openings per burst, burst durations, and P_open_ values within bursts, were minimally altered by either SHE-associated mutation

Our final sets of comparisons were for properties describing the small and large amplitude bursts of LS-isoform α4β2-nAChR harboring SHE-associated mutant subunits. We began by dividing open events into either single isolated openings, or bursts (defined as two or more openings separated by less than a critical closed time, see *Experimental Procedures* for details). This allowed us to determine the proportion of bursts within the population of all events (the sum of individual isolated openings and bursts). Then, within the open events defined as bursts, we measured the mean numbers of openings within bursts, the durations of these bursts, and the proportion of time spent open within bursts.

**Fig 9:**
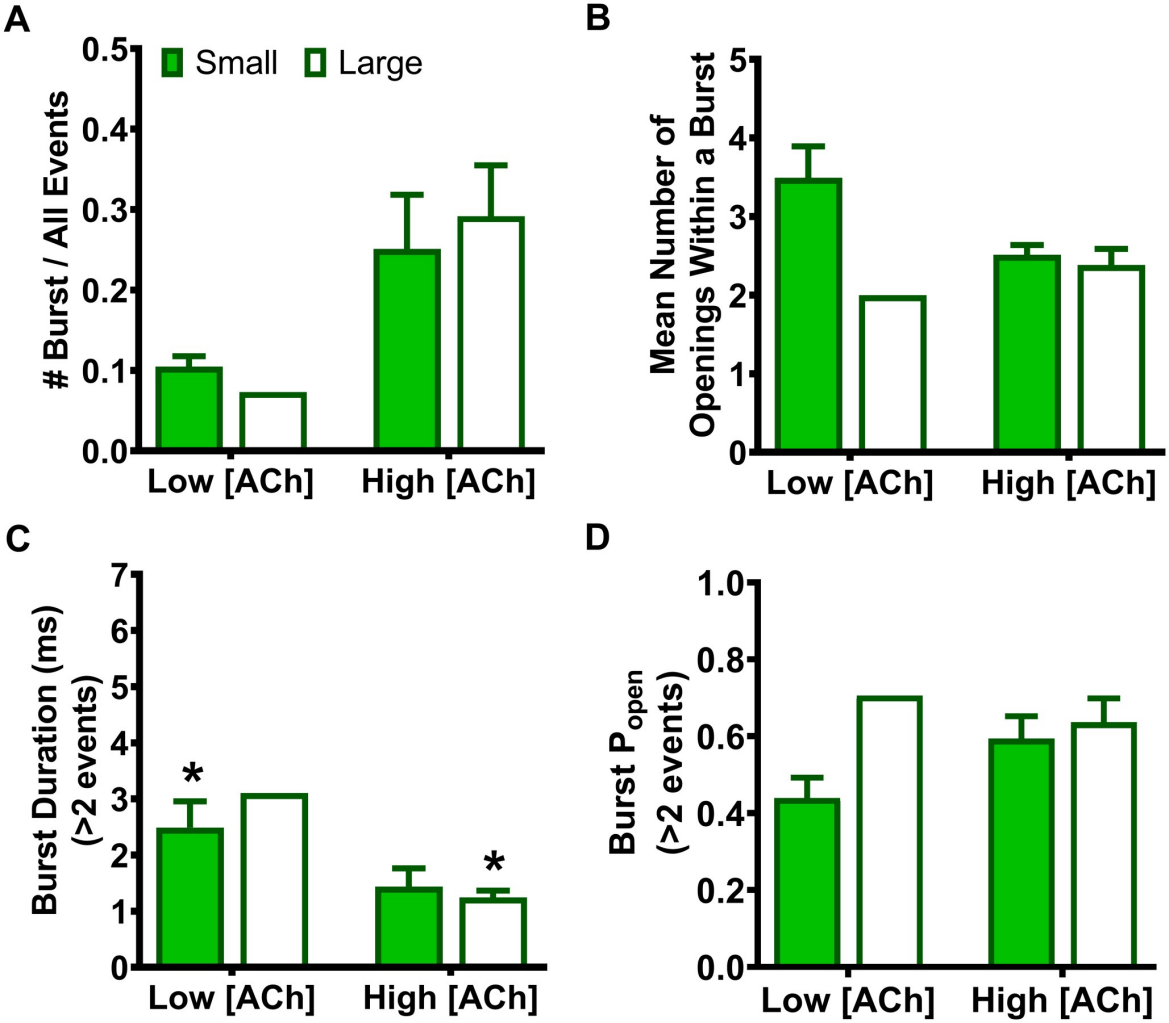
Human LS-isoform α4(R336H)β2-nAChR small and large amplitude burst properties. LS-isoform α4(R336H)β2-nAChR were expressed in *Xenopus laevis* oocytes. Function was evoked using 0.7 or 30 μM ACh and properties of the resulting bursts of open events were measured. (A) The proportions of small or large amplitude openings falling within bursts (as a fraction of the total numbers of each type of open event) were determined. (B) The mean number of openings per burst of LS-isoform α4(R336H)β2-nAChR was determined. (C) The durations of LS-isoform α4(R336H)β2-nAChR small or large amplitude bursts were measured at the low (0.7 μM) and high (30 μM) ACh concentrations. Noted statistical differences refer to the difference between the receptor property measured for LS-isoform α4(R336H)β2-nAChR versus its wildtype counterpart (i.e. fold change). (D) The P_open_ within bursts was also measured for bursts of small or large amplitude openings evoked from LS-isoform α4(R336H)β2-nAChR in the presence of the low or high ACh concentrations. Histograms within each panel show pooled data which were collected from 5 or 8 individual patches, respectively, across at least three separate experiments. Values and error bars represent the mean ± SEM of values across patches, and are summarized in Table 7, along with the statistical analyses applied.

We began by examining effects of α4(R336H) subunit incorporation into LS-isoform α4β2-nAChR. The proportion of bursts, overall, was low for LS-isoform α4(R336H)β2-nAChR, for either small or large amplitude openings, and at either ACh concentration (Fig 9A, Table 7). This indicates that most events occur as single openings in all conditions examined, a finding that matches that of our earlier study of wildtype human α4β2-nAChR [25]. Indeed, when stimulated at the lower (0.7 μM) ACh concentration, the bursting properties (proportion of bursts, mean number of events per burst, and open probability), for both classes of bursts, were mostly indistinguishable from those previously measured from wildtype α4β2-nAChR (Fig 9, Table 7). Only in one case was a significant change observed: the duration of bursts of small amplitude openings was reduced by approximately 30% when compared to LS-isoform wildtype α4β2-nAChR stimulated at the same concentration (Fig 9C, Table 7). When LS-isoform α4(R336H)β2-nAChR were stimulated with the higher (30 μM) ACh concentration, essentially the same outcome was observed—only one property was significantly different from that measured from wildtype α4β2-nAChR, again for burst durations, but in this case a 30% reduction in the length of bursts of large amplitude openings was observed when compared to the outcome in wildtype α4β2-nAChR (Fig 9C, Table 7).

**Table 7:** Single-channel properties associated with bursts of LS-isoform α4(R336H)β2-nAChR.

Isoform	#Burst/All Events		Mean Number of Openings Within a Burst		Burst Duration ± SEM (ms)		P_open_	
	Small	Large	Small	Large	Small	Large	Small	Large
*Low [ACh] (0*.*7 μM)*								
α4(R336H)β2 LS	0.10 ± 0.01	0.073	3.5 ± 0.4	2	2.5 ± 0.5	3.1	0.44 ± 0.05	0.7
Fold Change ± SEM	1.3 ± 0.2	0.9	1.5 ± 0.2	0.8	0.7 ± 0.1*	0.7	1.1 ± 0.3	1.2
*High [ACh] (30 μM)*								
α4(R336H)β2 LS	0.25 ± 0.07	0.29 ± 0.06	2.5 ± 0.1^‡^	2.4 ± 0.2	1.4 ± 0.3	1.2 ± 0.1	0.59 ± 0.06	0.64 ± 0.06
Fold Change ± SEM	1.1 ± 0.3	1.5 ± 0.3	1.0 ± 0.1	1.0 ± 0.1	1.4 ± 0.4	0.7 ± 0.1*	1.1 ± 0.1	1.0 ± 0.1

Single-channel properties associated with bursts of LS-isoform α4(R336H)β2-nAChR openings are illustrated in Fig 9. Data shown in this table are mean ± SEM of the properties derived from five (low ACh concentration; 0.7 μM) or eight (high ACh concentration; 30 μM) individual patches. The mean value of each property was compared to its counterpart, determined in parallel and under identical conditions, from wildtype LS-isoform α4β2-nAChR, using a One Sample T-test as detailed in the *Experimental Procedures* section. In each case, the fold difference relative to the corresponding property of the wildtype α4β2-nAChR counterpart is reported as Fold Change ± SEM. Most burst-associated properties were unaffected by introduction of the α4(R336H) SHE-associated mutant subunit, although two exceptions are noted. The durations of small amplitude bursts were shortened, but only when stimulated with the low ACh concentration (* P < 0.05, t = 3.00, df = 4). In contrast, durations of large amplitude bursts were lengthened, but only when evoked using the high ACh concentration (* P < 0.05, t = 3.00, df = 7). No other significant differences were observed.

**Fig 10:**
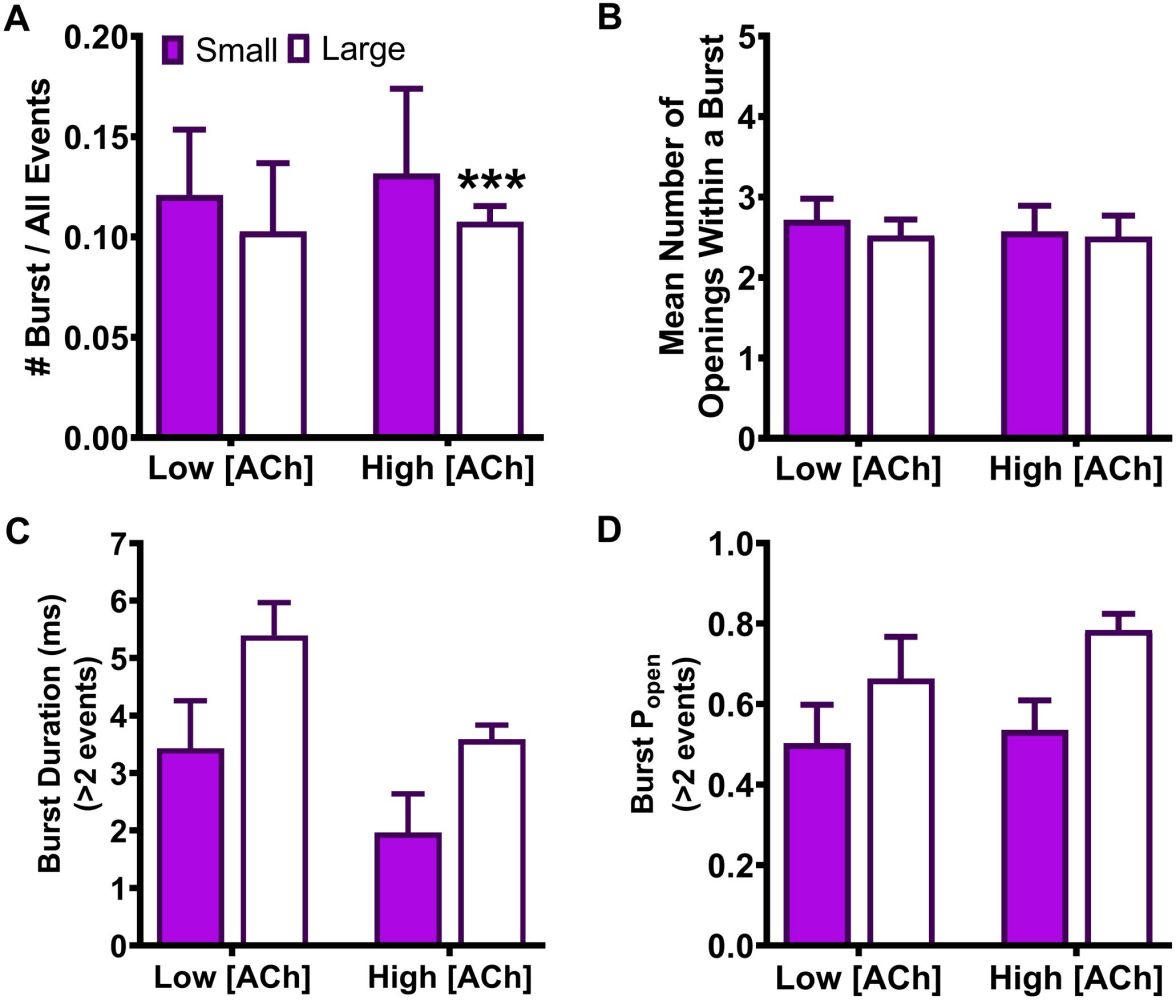
Human LS-isoform α4β2(V337G)-nAChR burst properties. LS-isoform α4β2(V337G)-nAChR were expressed in *Xenopus laevis* oocytes. Function was evoked using 0.7 or 30 μM ACh, and properties of the resulting bursts of open events were measured. (A) The proportions of small or large amplitude openings falling within bursts (as a fraction of the total numbers of each type of open event) were determined. Noted statistical difference refers to the difference between the receptor property measured for the LS-isoform α4β2(V337G)-nAChR versus its wildtype counterpart. (B) The mean number of openings per burst of LS-isoform α4β2(V337G)-nAChR were measured at each of the two ACh concentrations, for bursts of small or large amplitude openings. (C) The durations of LS-isoform α4β2(V337G)-nAChR small or large amplitude bursts were measured at the low (0.7 μM) and high (30 μM) ACh concentrations. (D) The P_open_ within bursts was also measured for bursts of small or large amplitude openings evoked from LS-isoform α4β2(V337G)-nAChR in the presence of the low or high ACh concentrations. Histograms within each panel show pooled data, which were collected from 5 and 7 individual patches respectively, across at least three separate experiments. Values and error bars represent the mean ± SEM of values across patches, and are summarized in Table 8, along with the statistical analyses applied.

Finally, we probed for possible effects of β2(V337G) mutant subunit incorporation into LS-isoform α4β2-nAChR. When responses were stimulated by the lower (0.7 μM) ACh concentration, all bursting properties, for both the small and large amplitude classes of bursts, were statistically indistinguishable from those measured from wildtype LS-isoform α4β2-nAChR within a burst (Fig 10, Table 8). The same outcome was observed when LS-isoform α4β2(V337G)-nAChR were stimulated with the higher (30 μM) ACh concentration, with only one exception: the proportion of bursts within overall large amplitude events was reduced by approximately 46% when compared to wildtype LS-isoform α4β2-nAChR (Fig 10A). We note a similar trend for the proportion of small amplitude events falling within a burst at the same ACh concentration, but this did not reach significance (also Fig 10A; P > 0.1, t = 2.28, df = 6; One Sample T-test). All other bursting properties, for both the small and large amplitude classes of bursts, were again statistically indistinguishable from those measured from wildtype LS-isoform α4β2-nAChR.

**Table 8:** Single-Channel properties associated with bursts of LS-isoform α4β2(V337G)-nAChR.

Isoform	#Burst/All Events		Mean Number of Openings Within a Burst		Burst Duration ± SEM (ms)		P_open_	
	Small	Large	Small	Large	Small	Large	Small	Large
*Low [ACh] (0*.*7 μM)*								
α4β2(V337G) LS	0.12 ± 0.03	0.10 ± 0.03	2.7 ± 0.3	2.5 ± 0.2	3.4 ± 0.8	5.4 ± 0.6	0.50 ± 0.10	0.7 ± 0.1
Fold Change ± SEM	0.2 ± 0.4	1.3 ± 0.4	1.1 ± 0.1	1.0 ± 0.1	1.0 ± 0.2	1.2 ± 0.1	0.9 ± 0.2	1.0 ± 0.2
*High [ACh] (30 μM)*								
α4β2(V337G) LS	0.13 ± 0.04	0.11 ± 0.01	2.6 ± 0.3	2.5 ± 0.3	2.0 ± 0.7	3.6 ± 0.2	0.53 ± 0.07	0.78 ± 0.04
Fold Change ± SEM	0.6 ± 0.2	0.56 ± 0.04***	1.0 ± 0.2	1.0 ± 0.1	1.4 ± 0.4	1.3 ± 0.1	1.0 ± 0.1	1.2 ± 0.1

In summary, burst analysis showed that very few properties are altered for either SHE mutation in comparison to their wildtype receptor counterparts. These changes in burst-associated properties are unlikely to contribute to the previously observed SHE mutant-driven increases in macroscopic function-per-receptor [14].

Single-channel properties associated with bursts of LS-isoform α4β2(V337G)-nAChR openings are shown in Fig 10. Data summarized in this table are mean ± SEM of the properties derived from five (low ACh concentration; 0.7 μM) or seven (high ACh concentration; 30 μM) individual patches. The mean value of each property was compared to its counterpart, determined in parallel and under identical conditions, from wildtype LS-isoform α4β2-nAChR, using a One Sample T-test as detailed in the *Experimental Procedures* section. In each case, the fold difference relative to the corresponding property of the wildtype α4β2-nAChR counterpart is reported as Fold Change ± SEM. As was the case for α4(R336H)β2-nAChR, almost no changes in burst-associated properties were induced by incorporation of the β2(V337G) SHE-associated mutant subunit. Only one exception was identified: when large amplitude events were evoked with the high ACh concentration, the proportion of these events falling within bursts was significantly reduced (*** P < 0.001, t = 10.70, df = 6).

## Discussion

**Fig 11:**
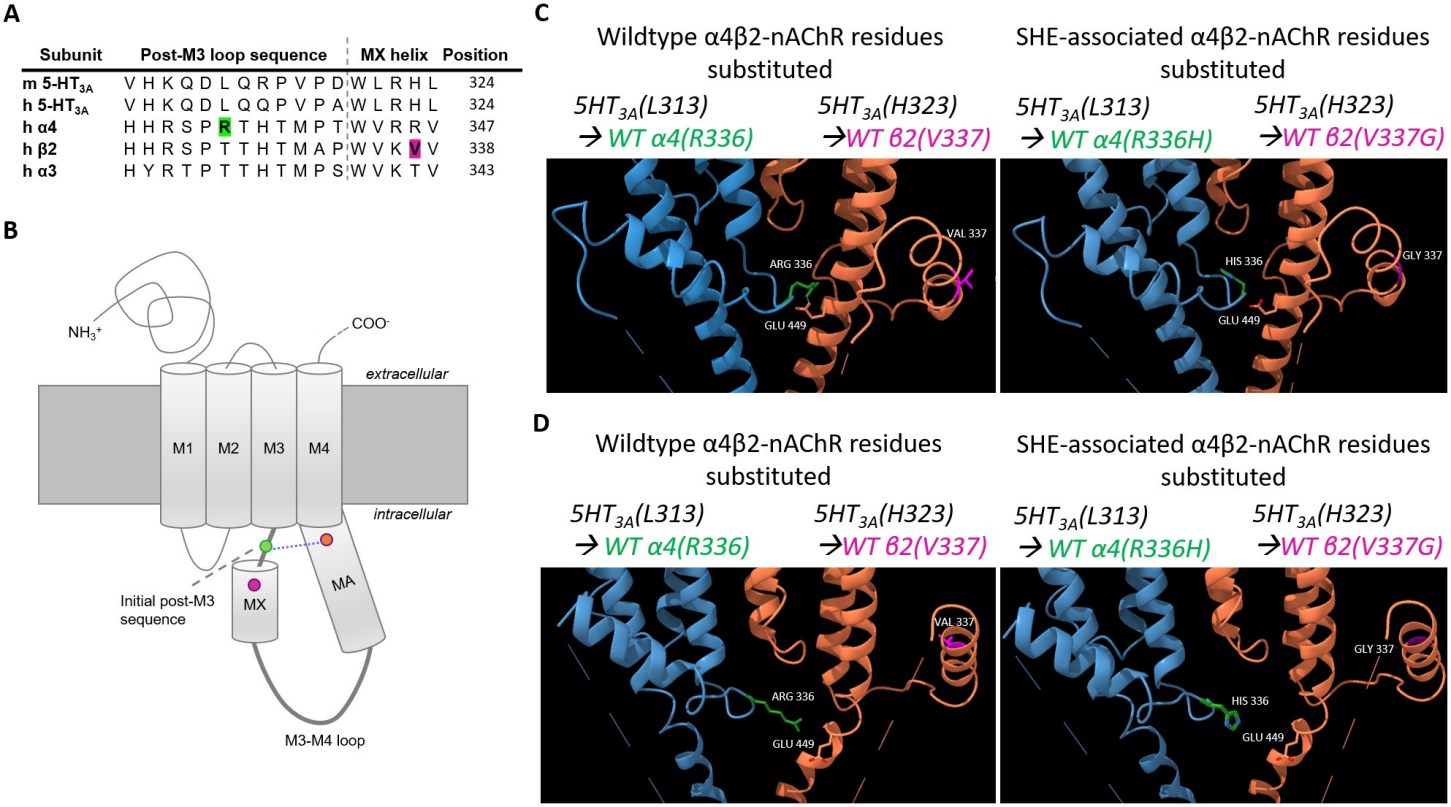
Illustration of the α4(R336H)β2- and α4β2(V337G)-nAChR SHE-associated mutant locations substituted into the known cryo-EM structures of 5-HT_3A_R apo and conducting states. (A) Sequence alignments of 5-HT_3A_R and nAChR subunits are displayed with numbering based on a start at the translation initiation methionine. The locations of the intracellular loop SHE-associated mutations are highlighted. The α4(R336H) mutation (green) is located in the initial portion of the M3-M4 loop, whereas the β2(V337G) mutation (magenta) is located in the MX helix. (B) Schematic of the major structural domains of a single nAChR subunit. The large extracellular N-terminal agonist-binding domain is followed by four transmembrane helices (M1–M4). The major intracellular domain is located between the M3 and M4 helices and consists of a short initial portion of sequence that connects the M3 helix to the MX helix, the MX helix itself, an extended region of undefined structure, followed by the final M4 transmembrane helix. The locations of both SHE-associated mutations are shown in the initial portion of the M3-M4 domain and the MX helix subdomain of the major intracellular domain, along with that of a likely interacting residue in the MA helix (β2E449; orange, putative interaction denoted with a dotted blue line). Note that β2E449 is not shown in Panel A since it is located far in the linear sequence from the MX helix. (C) The apo (non-ligand-bound; PDB ID: 6BE1) state of the recent cryo-EM structure of the 5-HT_3A_R [45] was used as the basis to illustrate the locations of the α4(R336H)β2- and α4β2(V337G)-nAChR mutations, since equivalent structural information is not yet available for these intracellular regions of α4β2-nAChR. Position numbers refer to those of the human α4 or β2 nAChR subunit, as shown in the accompanying sequence alignment. *Left Panel* depicts the positioning of wildtype nAChR α4(R336) and β2(V337) residues, substituted into the known 5-HT_3A_R structure. The location of the initial portion of the M3-M4 domain, followed by the MX, MA, and M4 helices are labeled. *Right Panel* illustrates the locations of the SHE-associated α4(R336H) and β2(V337G) residues in the same context. Also shown in both panels is the location of the β2E449 residue thought to interact with α4(R336). (D) Same as (C), except that the residues of interest are substituted into the conducting (ligand-bound; PDB ID: 6DG8) 5-HT_3A_R cryo-EM structure [31]. Note the upward movement of the MX helix in the conducting, compared to apo, state.

Here, we use a single-channel patch clamp electrophysiology approach to define for the first time the specific unitary mechanisms by which nAChR α4(R336H) and β2(V337G) subunit mutations alter α4β2-nAChR function. Several recent studies employing cryo-electron microscopy [31,32,45] provide valuable new ligand gated ion channel (LGIC) structural information for the regions hosting these two mutations, giving further context to our novel findings. The equivalent positions of these two mutations within the subunits’ linear sequences, along with that of one additional interacting residue, are illustrated in Fig 11. Fig 11 also shows these residues’ locations within the context of the recent 5HT_3A_R cryo-EM closed (apo) and open (conducting) structures [31,45].

The present study shows that introduction of the α4(R336H) SHE-associated mutant subunit has only minor effects on the amplitude of single-channel openings, or on the properties of bursts of such openings, of either HS- or LS-isoform α4β2-nAChR. When considering the durations of individual openings, the situation appears, at first glance, to be similar. When all openings are considered (both within and outside of bursts) neither the lengths of openings of HS-, nor of LS-isoform α4β2-nAChR, are altered. However, as shown in our previous work [25], this broad analysis can be deceiving in the case of LS-isoform α4β2-nAChR since LS-isoform α4β2-nAChR bursts are segregated into repeated small or large amplitude openings, not a mixture of both). A segregated analysis of the two burst classes uncovered a bias towards HS-isoform function induced by α4(R336H) subunit incorporation. As detailed in the results section, at the lower ACh concentration applied, small amplitude openings were shorter than those seen for wildtype LS-isoform α4β2-nAChR, and large amplitude openings were abolished in all but one of the recorded patches. At the higher ACh concentration, the α4(R336H) LS-isoform small amplitude openings remained shorter, whereas large amplitude openings returned and were longer in duration, than those recorded from wildtype α4β2-nAChR. Under physiological conditions where expression of a mixed population of HS- and LS-isoform α4(R336H)β2-nAChR occurs [17], the overall macroscopic effect would be to reduce LS-isoform function compared to HS-isoform function, at least at lower ACh concentrations.

The most striking and consistent effect of incorporating the α4(R336H) subunit was noted with analysis of closed-dwell times. Closed times between openings of HS-isoform α4β2-nAChR were significantly shortened, as were those between bursts of LS-isoform function (of either amplitude class). The number of receptors per patch can significantly affect closed durations between openings and bursts, but note that surface expression of α4(R336H)β2-nAChR or α4β2(V337G)-nAChR is not different to that of wildtype α4β2-nAChR under the same conditions applied here [14]. Further, we applied quality-control criteria to remove from our analysis any patches with atypically high (or low) expression levels (see *Experimental Procedures)*. Long closed times between bursts correspond to desensitized periods [46,47]. Accordingly, our results indicate that the previously-noted increase in per-receptor-function of α4(R336H)β2-nAChR is primarily due to destabilization of desensitized states. Additionally, no effect was observed on the open probability of bursts recorded from α4(R336H)β2-nAChR, and the overall pattern of α4(R336H)β2-nAChR activation remained sparse. These results indicate a low open probability for α4β2-nAChR, even in the presence of this SHE-associated mutant subunit.

Based on cryo-EM structures of the 5-HT_3A_R in apo and ion-conducting states, the α4R336 residue (equivalent to the 5-HT_3A_L313 residue; Fig 11C and 11D) is located between the M3 and the MX helices [31,45]. In the apo state, residues within the post-M3 sequence obstruct the lateral portals lined by the MA-M4 helices. In the ion conducting state, however, this initial portion of the M3-M4 loop preceding the MX domain extends away from the MA and M4 helices, creating lateral portals large enough to fit hydrated Na^+^ ions, allowing ion permeation. Further, a α3β4-nAChR cryo-EM study identified that residue α3T303 (located within the initial portion of the M3-M4 loop preceding the MX helix) and the MA helix β4E428 residue (Fig 11C and 11D) form a hydrogen bond in the desensitized state [32]. These positions correspond in wildtype α4β2-nAChR to α4R336 and β2E449, respectively, and so would be expected to form a favorable electrostatic interaction in these equivalent positions of a desensitized wildtype α4β2-nAChR. In this context, an α4(R336H) mutation would be expected to alter such an electrostatic interaction, and potentially contribute to the observed destabilization of desensitized states.

Outside of a similar lack of effect on properties of LS-isoform bursts, the consequences of the β2(V337G) mutation were quite different to those of the just-discussed α4(R336H) mutation. One striking difference was that introduction of β2(V337G) subunit reduced the amplitudes of openings of HS- and LS-isoform α4β2(V337G)-nAChR. This was true for all amplitude classes and across all ACh concentrations and, logically, would not produce increased per-receptor function. Intriguingly, the effect was most-pronounced for the HS-isoform (which contains three β2 subunits, compared to only two in the LS-isoform). This suggests the possibility of a subunit protein stoichiometry effect. As a possible extension of this subunit protein stoichiometry concept the only effect of the α4(R336H) subunit that we observed (on open-dwell durations within bursts) was in the context of the LS-isoform which contains three α4 subunits, compared to only two in the HS-isoforms.

Closed-dwell time analysis showed complex effects of β2(V337G) subunit incorporation. No effect was seen on HS-isoform α4β2(V337G)-nAChR. Analysis of all closed-dwell times between openings at the lower ACh concentration for LS-isoform α4β2(V337G)-nAChR showed that they were shortened compared to wildtype α4β2-nAChR (thereby increasing overall function). However, this finding was reversed (lengthened closed times, thereby decreasing overall function) at the higher ACh concentration. Ultimately this would be expected to increase function of LS-isoform nAChR disproportionately at low ACh concentrations (i.e. within the HS-phase of function). In a mixed population of HS- and LS-isoform nAChR that occurs under physiological conditions, and likely in neurons expressing SHE-associated nAChR subunits, the closed-dwell times effects would result in a higher proportion of HS-phase function overall. This all events analysis was largely confirmed by the more-detailed analysis of segregated small and large amplitude bursts of LS-isoform α4β2(V337G)-nAChR openings. At the higher ACh concentration, closed-dwell times between both small and large amplitude bursts were extended compared to their wildtype counterparts, a finding that agrees with the analysis inclusive of all events. At the lower ACh concentration, closed-dwell times between small amplitude bursts were also significantly extended compared to those measured for WT α4β2-nAChR. However, closed-dwell time durations between large amplitude bursts were shortened resulting in an increase in overall function. This effect presumably predominated the all events analysis. This provides an excellent example of how subtle, but meaningful, differences hidden within the overall analysis can be uncovered when it is possible to reliably segregate effects between/across bursts of different amplitudes.

We previously showed that a major effect of agonist engagement of the lower-affinity α4/α4 agonist-binding site by higher ACh concentrations is to shorten closed times between bursts of openings of wildtype LS-isoform α4β2-nAChR [25]. Examining the absolute values of closed-dwell times between single-channel bursts of each class for LS-isoform α4β2(V337G)-nAChR (Table 5) is instructive. For bursts with small amplitudes, engagement of the α4/α4 site at the higher ACh concentration shortens each of the interburst intervals τ_S1_, τ_S2_, and τ_S3_, but to a lesser extent than in wildtype receptors. For bursts with large amplitudes, the effect is reversed–agonist engagement of the α4/α4 site actually extended closed-dwell times between large amplitude bursts (τ_L1_, τ_L2_, and τ_L3_). These findings provide consistent evidence that the β2(V337G) mutation interacts with the operation of the α4/α4 site of LS-isoform α4β2(V337G)-nAChR. In turn, this may indicate that the 337 position is part of a mechanistic link between agonist binding at the α4/α4 site and destabilization of desensitized states of LS-isoform α4β2-nAChR.

However, the most consistent and dramatic effects of incorporating the β2(V337G) subunit were seen on open-dwell times of LS-isoform α4β2(V337G)-nAChR. For wildtype α4β2-nAChR, open-dwell times of LS-isoform single-channel events are best described with a single time constant for both ACh concentrations applied, and for bursts of both the small or large amplitude classes [25]. Activity of LS-isoform α4β2(V337G)-nAChR was largely consistent with this: open-dwell times of both small and large amplitude bursts of activity at the higher ACh concentration, and of large amplitude bursts at the lower ACh concentration, were each best fit with single time constants (albeit significantly extended compared to their wildtype counterparts). However, the outcomes for small amplitude openings in the presence of the low ACh concentration were more complicated. In this case, two different open-dwell times were observed; one significantly shorter, and the other significantly longer, than the single duration measured for the wildtype LS-isoform under the same condition. If we hypothesize that the general effect of the β2(V337G) mutation is to prolong openings, it is possible that the emergence of a very short open-dwell time population under these conditions may reflect uncovering of a population of openings that, in wildtype α4β2-nAChR, is too transient to be observed under our filtering and data-processing protocol. If so, one could speculate that the shorter activation events at the lower ACh concentration might be caused by activation through a single canonical agonist binding site, while the longer-lived openings might be caused by activation via both canonical binding sites, as was suggested for muscle-type nAChR [48]. In the case of the muscle-type nAChR, this hypothesis was later disproved since “If this were the case, brief openings should become relatively less abundant as the agonist concentration is raised” [49], and such a concentration-dependent effect was not seen. However, our current study shows that only longer openings are observed at the higher ACh concentration, which would be compatible with a concentration-dependent effect. Regardless of the precise functional interaction, β2(V337G) subunit incorporation appears generally to prolong open-dwell times. As such, this effect on open-dwell times must counteract the smaller open-event amplitudes associated with α4β2(V337G)-nAChR, thereby being responsible for the overall increase in per-receptor function noted during our earlier macroscopic study [14].

The β2(V337G) SHE-associated mutation is located within the MX helix, which lies parallel to the putative membrane-water interface of the apo (closed-state) 5-HT_3A_R cryo-EM structure [31,45]. In the ion conducting state, the MX helix embeds within the lipid bilayer, pulling the initial portion of the M3-M4 that precedes the MX helix loop away from the lateral portals, helping to create openings for ions to pass [31] (see Fig 11C and 11D). A hydrophobic valine residue in the MX helix would be favorable as this sidechain can interact strongly with the terminal tails of lipids in the lipid membrane core [50]. However, incorporation of the β2(V337G) mutant would be predicted to remove these tail interactions, and the glycine backbone would instead be free to form hydrogen bonds with lipid phosphate groups [51]. Further, introduction of a highly-flexible glycine residue is not favorable to helix formation, and could destabilize the MX helix. Together, these potential alterations by the β2(V337G) mutation could modify the transitions between open and closed conformations of the host α4β2(V337G)-nAChR giving rise to the kinetic effects noted in our single channel analysis. In addition, [31] points out that rearrangement of the MX helix is coupled to changes in the alignment of the MA and M4 helices. This movement of the MA helix is particularly important since it plays a major part in opening ion permeation pores in the open state and repositions residues known to influence receptor conduction through these pores [31]. It is possible that the altered open channel amplitudes resulting from incorporation of the β2(V337G) mutation may be due to its ability to alter the precise positioning of the MA helix during this set of coordinated conformational changes. Conformation of these hypotheses would require an extensive new study in which further mutations were made, alongside molecular dynamics modeling of membrane lipid-receptor interactions and ion permeation rates.

Overall, this study demonstrates that functional changes induced by the α4(R336H) and β2(V337G) subunits are complex, but some general rules emerge. Increases in macroscopic function appear to be driven primarily by alterations in single-channel kinetic properties, rather than unitary amplitudes. For the α4(R336H) mutation, destabilization of receptor desensitized states appears to be the predominant effect, while the principal factor in the case of the β2(V337G) mutation is stabilization of open states. Considering these single-channel outcomes in the context of recent structural biology discoveries provides valuable context and suggests possible mechanistic bases for our functional observations. These mutually-supporting studies also reinforce the concept that intracellular structures of Cys-loop receptors play physiologically-important roles in determining kinetics of channel opening and desensitization [52,53]. Especially since the precise phenomena underlying Cys-loop receptor desensitization remain somewhat mysterious, this is an important contribution.

From a clinical perspective, our findings support the conclusion of our earlier publication: that the increase in α4β2-nAChR function likely results in an enhancement of neuronal excitability, causing an imbalance between inhibitory and excitatory synaptic transmission, leading to seizures [14]. More-generally, given the wide range of influences of nAChR on normal and disease physiology [54–58], novel mechanistic insights can have valuable translational applications (such as defining what effects of a given mutation need to be ameliorated to restore normal physiological function). The use of naturally-occurring and physiologically-impactful mutations has given us an opportunity to define new and valuable information about how the important class of Cys-loop receptors functions.

## Supporting information

S1 Data Measured values for unitary amplitudes, closed dwell times, open dwell times, event frequencies, and Popen. Excel file contains single-channel data for SHE-containing α4β2 nAChR single-channel parameters calculated using QuB for each patch. Averages, S.E.M, and outlier test calculations are included.
(XLSX)

S2 Data Pooled interburst duration raw data values. Raw data values for each patch for SHE-containing α4β2-nAChR as measured using QuB. Data for each patch has been pooled into a single column. LS-isoform data has been separated by event amplitude class.
(XLSX)

S3 Data Pooled intraburst duration raw data values. Raw data values for each patch for SHE-containing α4β2-nAChR as measured by QuB. Data for each patch has been pooled into a single column. LS-isoform data has been separated by event amplitude class.
(XLSX)

S4 Data The number of events evoked with a burst for SHE-containing α4β2-nAChR. Raw data were determined using QuB, and LS α4β2-nAChR data are separated by amplitude class designation.
(XLSX)

S5 Data Burst duration values for SHE-containing α4β2-nAChR. Raw data values were measured using QuB. LS-isoform SHE-containing α4β2-nAChR data are separated by amplitude class designation.
(XLSX)

## References

[1] Tinuper P, Bisulli F, Cross JH, Hesdorffer D, Kahane P, Nobili L, et al. Definition and diagnostic criteria of sleep-related hypermotor epilepsy. Neurology. 2016;86(19):1834–42. 10.1212/WNL.0000000000002666 27164717 PMC4862248

[2] Scheffer IE, Bhatia KP, Lopes-Cendes I, Fish DR, Marsden CD, Andermann F, et al. Autosomal dominant frontal epilepsy misdiagnosed as sleep disorder. Lancet. 1994;343(8896):515–7. 10.1016/s0140-6736(94)91463-x 7906762

[3] Steinlein OK, Mulley JC, Propping P, Wallace RH, Phillips HA, Sutherland GR, et al. A missense mutation in the neuronal nicotinic acetylcholine receptor alpha 4 subunit is associated with autosomal dominant nocturnal frontal lobe epilepsy. Nat Genet. 1995;11(2):201–3. 10.1038/ng1095-201 7550350

[4] Lukas RJ, Changeux JP, Le Novere N, Albuquerque EX, Balfour DJ, Berg DK, et al. International Union of Pharmacology. XX. Current status of the nomenclature for nicotinic acetylcholine receptors and their subunits. Pharmacol Rev. 1999;51(2):397–401. 10353988

[5] Zoli M, Pucci S, Vilella A, Gotti C. Neuronal and Extraneuronal Nicotinic Acetylcholine Receptors. Curr Neuropharmacol. 2018;16(4):338–49. 10.2174/1570159X15666170912110450 28901280 PMC6018187

[6] Bertrand D, Elmslie F, Hughes E, Trounce J, Sander T, Bertrand S, et al. The CHRNB2 mutation I312M is associated with epilepsy and distinct memory deficits. Neurobiol Dis. 2005;20(3):799–804. 10.1016/j.nbd.2005.05.013 15964197

[7] De Fusco M, Becchetti A, Patrignani A, Annesi G, Gambardella A, Quattrone A, et al. The nicotinic receptor beta 2 subunit is mutant in nocturnal frontal lobe epilepsy. Nat Genet. 2000;26(3):275–6. 10.1038/81566 11062464

[8] Hirose S, Iwata H, Akiyoshi H, Kobayashi K, Ito M, Wada K, et al. A novel mutation of CHRNA4 responsible for autosomal dominant nocturnal frontal lobe epilepsy. Neurology. 1999;53(8):1749–53. 10.1212/wnl.53.8.1749 10563623

[9] Steinlein OK. Genetic mechanisms that underlie epilepsy. Nat Rev Neurosci. 2004;5(5):400–8. 10.1038/nrn1388 15100722

[10] Steinlein OK. Animal models for autosomal dominant frontal lobe epilepsy: on the origin of seizures. Expert Rev Neurother. 2010;10(12):1859–67. 10.1586/ern.10.130 21091316

[11] Steinlein OK, Magnusson A, Stoodt J, Bertrand S, Weiland S, Berkovic SF, et al. An insertion mutation of the CHRNA4 gene in a family with autosomal dominant nocturnal frontal lobe epilepsy. Hum Mol Genet. 1997;6(6):943–7. 10.1093/hmg/6.6.943 9175743

[12] Chen Y, Wu L, Fang Y, He Z, Peng B, Shen Y, et al. A novel mutation of the nicotinic acetylcholine receptor gene CHRNA4 in sporadic nocturnal frontal lobe epilepsy. Epilepsy Res. 2009;83(2–3):152–6. 10.1016/j.eplepsyres.2008.10.009 19058950

[13] Liu H, Lu C, Li Z, Zhou S, Li X, Ji L, et al. The identification of a novel mutation of nicotinic acetylcholine receptor gene CHRNB2 in a Chinese patient: Its possible implication in non-familial nocturnal frontal lobe epilepsy. Epilepsy Res. 2011;95(1–2):94–9. 10.1016/j.eplepsyres.2011.03.002 21497487

[14] Weltzin MM, Lindstrom JM, Lukas RJ, Whiteaker P. Distinctive effects of nicotinic receptor intracellular-loop mutations associated with nocturnal frontal lobe epilepsy. Neuropharmacology. 2016;102:158–73. 10.1016/j.neuropharm.2015.11.004 26561946 PMC4698238

[15] Briggs CA, Gubbins EJ, Putman CB, Thimmapaya R, Meyer MD, Surowy CS. High- and low-sensitivity subforms of alpha4beta2 and alpha3beta2 nAChRs. J Mol Neurosci. 2006;30(1–2):11–2. 10.1385/JMN:30:1:11 17192606

[16] Eaton JB, Lucero LM, Stratton H, Chang Y, Cooper JF, Lindstrom JM, et al. The unique alpha4+/-alpha4 agonist binding site in (alpha4)3(beta2)2 subtype nicotinic acetylcholine receptors permits differential agonist desensitization pharmacology versus the (alpha4)2(beta2)3 subtype. J Pharmacol Exp Ther. 2014;348(1):46–58. 10.1124/jpet.113.208389 24190916 PMC3868879

[17] Marks MJ, Whiteaker P, Calcaterra J, Stitzel JA, Bullock AE, Grady SR, et al. Two pharmacologically distinct components of nicotinic receptor-mediated rubidium efflux in mouse brain require the beta2 subunit. J Pharmacol Exp Ther. 1999;289(2):1090–103. 10215692

[18] Mazzaferro S, Benallegue N, Carbone A, Gasparri F, Vijayan R, Biggin PC, et al. Additional acetylcholine (ACh) binding site at alpha4/alpha4 interface of (alpha4beta2)2alpha4 nicotinic receptor influences agonist sensitivity. J Biol Chem. 2011;286(35):31043–54. 10.1074/jbc.M111.262014 21757735 PMC3162463

[19] Moroni M, Bermudez I. Stoichiometry and pharmacology of two human alpha4beta2 nicotinic receptor types. J Mol Neurosci. 2006;30(1–2):95–6. 10.1385/JMN:30:1:95 17192644

[20] Nelson ME, Kuryatov A, Choi CH, Zhou Y, Lindstrom J. Alternate stoichiometries of alpha4beta2 nicotinic acetylcholine receptors. Mol Pharmacol. 2003;63(2):332–41. 10.1124/mol.63.2.332 12527804

[21] Tapia L, Kuryatov A, Lindstrom J. Ca2+ permeability of the (alpha4)3(beta2)2 stoichiometry greatly exceeds that of (alpha4)2(beta2)3 human acetylcholine receptors. Mol Pharmacol. 2007;71(3):769–76. 10.1124/mol.106.030445 17132685

[22] Indurthi DC, Qudah T, Liao VW, Ahring PK, Lewis TM, Balle T, et al. Revisiting autosomal dominant nocturnal frontal lobe epilepsy (ADNFLE) mutations in the nicotinic acetylcholine receptor reveal an increase in efficacy regardless of stochiometry. Pharmacol Res. 2019;139:215–27. 10.1016/j.phrs.2018.11.031 30472464

[23] Kracun S, Harkness PC, Gibb AJ, Millar NS. Influence of the M3-M4 intracellular domain upon nicotinic acetylcholine receptor assembly, targeting and function. Br J Pharmacol. 2008;153(7):1474–84. 10.1038/sj.bjp.0707676 18204482 PMC2437914

[24] Hales TG, Dunlop JI, Deeb TZ, Carland JE, Kelley SP, Lambert JJ, et al. Common determinants of single channel conductance within the large cytoplasmic loop of 5-hydroxytryptamine type 3 and alpha4beta2 nicotinic acetylcholine receptors. J Biol Chem. 2006;281(12):8062–71. 10.1074/jbc.M513222200 16407231

[25] Weltzin MM, George AA, Lukas RJ, Whiteaker P. Distinctive single-channel properties of alpha4beta2-nicotinic acetylcholine receptor isoforms. PLoS One. 2019;14(3):e0213143. 10.1371/journal.pone.0213143 30845161 PMC6405073

[26] Mazzaferro S, Bermudez I, Sine SM. alpha4beta2 Nicotinic Acetylcholine Receptors: RELATIONSHIPS BETWEEN SUBUNIT STOICHIOMETRY AND FUNCTION AT THE SINGLE CHANNEL LEVEL. J Biol Chem. 2017;292(7):2729–40. 10.1074/jbc.M116.764183 28031459 PMC5314170

[27] Provini F, Plazzi G, Tinuper P, Vandi S, Lugaresi E, Montagna P. Nocturnal frontal lobe epilepsy. A clinical and polygraphic overview of 100 consecutive cases. Brain. 1999;122 (Pt 6):1017–31. 10.1093/brain/122.6.1017 10356056

[28] Motamedi GK, Lesser RP. Autosomal dominant nocturnal frontal lobe epilepsy. Advances in neurology. 2002;89:463–73. 11968471

[29] Kullmann DM. Genetics of epilepsy. J Neurol Neurosurg Psychiatry. 2002;73 Suppl 2:II32–5. 10.1136/jnnp.73.suppl_2.ii32 12536158 PMC1765606

[30] Walsh RM Jr., Roh SH, Gharpure A, Morales-Perez CL, Teng J, Hibbs RE. Structural principles of distinct assemblies of the human alpha4beta2 nicotinic receptor. Nature. 2018;557(7704):261–5. 10.1038/s41586-018-0081-7 29720657 PMC6132059

[31] Basak S, Gicheru Y, Rao S, Sansom MSP, Chakrapani S. Cryo-EM reveals two distinct serotonin-bound conformations of full-length 5-HT3A receptor. Nature. 2018;563(7730):270–4. 10.1038/s41586-018-0660-7 30401837 PMC6237196

[32] Gharpure A, Teng J, Zhuang Y, Noviello CM, Walsh RM Jr., Cabuco R, et al. Agonist Selectivity and Ion Permeation in the alpha3beta4 Ganglionic Nicotinic Receptor. Neuron. 2019;104(3):501–11.e6. 10.1016/j.neuron.2019.07.030 31488329 PMC6842111

[33] Eaton JB, Lucero LM, Stratton H, Chang Y, Cooper JF, Lindstrom JM, et al. The Unique α4(+)/(−)α4 Agonist Binding Site in (α4)3(β2)2 Subtype Nicotinic Acetylcholine Receptors Permits Differential Agonist Desensitization Pharmacology versus the (α4)2(β2)3 Subtype. Journal of Pharmacology and Experimental Therapeutics. 2014;348(1):46–58. 10.1124/jpet.113.208389 24190916 PMC3868879

[34] Papke RL, Oswald RE. Mechanisms of noncompetitive inhibition of acetylcholine-induced single-channel currents. J Gen Physiol. 1989;93(5):785–811. 10.1085/jgp.93.5.785 2472461 PMC2216236

[35] Qin F. Restoration of single-channel currents using the segmental k-means method based on hidden Markov modeling. Biophys J. 2004;86(3):1488–501. 10.1016/S0006-3495(04)74217-4 14990476 PMC1303984

[36] Qin F, Auerbach A, Sachs F. Maximum likelihood estimation of aggregated Markov processes. Proceedings Biological sciences/The Royal Society. 1997;264(1380):375–83. 10.1098/rspb.1997.0054 9107053 PMC1688268

[37] Qin F, Auerbach A, Sachs F. Estimating single-channel kinetic parameters from idealized patch-clamp data containing missed events. Biophys J. 1996;70(1):264–80. 10.1016/S0006-3495(96)79568-1 8770203 PMC1224925

[38] Carignano C, Barila EP, Spitzmaul G. Analysis of neuronal nicotinic acetylcholine receptor α4β2 activation at the single-channel level. Biochimica et Biophysica Acta (BBA)—Biomembranes. 2016;1858(9):1964–73. 10.1016/j.bbamem.2016.05.019 27233449

[39] Sakmann B, Patlak J, Neher E. Single acetylcholine-activated channels show burst-kinetics in presence of desensitizing concentrations of agonist. Nature. 1980;286(5768):71–3. 10.1038/286071a0 6248795

[40] Jackson MB, Wong BS, Morris CE, Lecar H, Christian CN. Successive openings of the same acetylcholine receptor channel are correlated in open time. Biophys J. 1983;42(1):109–14. 10.1016/S0006-3495(83)84375-6 6301575 PMC1329209

[41] Militante J, Ma BW, Akk G, Steinbach JH. Activation and block of the adult muscle-type nicotinic receptor by physostigmine: single-channel studies. Mol Pharmacol. 2008;74(3):764–76. 10.1124/mol.108.047134 18523135 PMC2536770

[42] Grosman C, Auerbach A. The dissociation of acetylcholine from open nicotinic receptor channels. Proc Natl Acad Sci U S A. 2001;98(24):14102–7. 10.1073/pnas.251402498 11717464 PMC61175

[43] Jadey S, Auerbach A. An integrated catch-and-hold mechanism activates nicotinic acetylcholine receptors. J Gen Physiol. 2012;140(1):17–28. 10.1085/jgp.201210801 22732309 PMC3382718

[44] Mortensen M, Smart TG. Single-channel recording of ligand-gated ion channels. Nat Protoc. 2007;2(11):2826–41. 10.1038/nprot.2007.403 18007618

[45] Basak S, Gicheru Y, Samanta A, Molugu SK, Huang W, Fuente M, et al. Cryo-EM structure of 5-HT3A receptor in its resting conformation. Nat Commun. 2018;9(1):514. 10.1038/s41467-018-02997-4 29410406 PMC5802770

[46] Katz B, Thesleff S. A study of the desensitization produced by acetylcholine at the motor end-plate. J Physiol. 1957;138(1):63–80. 10.1113/jphysiol.1957.sp005838 13463799 PMC1363030

[47] Bouzat C, Barrantes FJ. Modulation of muscle nicotinic acetylcholine receptors by the glucocorticoid hydrocortisone. Possible allosteric mechanism of channel blockade. J Biol Chem. 1996;271(42):25835–41. 10.1074/jbc.271.42.25835 8824214

[48] Colquhoun D, Sakmann B. Fluctuations in the microsecond time range of the current through single acetylcholine receptor ion channels. Nature. 1981;294(5840):464–6. 10.1038/294464a0 6273743

[49] Sine SM, Steinbach JH. Activation of a nicotinic acetylcholine receptor. Biophys J. 1984;45(1):175–85. 10.1016/S0006-3495(84)84146-6 6324901 PMC1435244

[50] Newport TD, Sansom MSP, Stansfeld PJ. The MemProtMD database: a resource for membrane-embedded protein structures and their lipid interactions. Nucleic Acids Res. 2019;47(D1):D390–D7. 10.1093/nar/gky1047 30418645 PMC6324062

[51] Porasso RD, Ale NM, Ciocco Aloia F, Masone D, Del Pópolo MG, Ben Altabef A, et al. Interaction of glycine, lysine, proline and histidine with dipalmitoylphosphatidylcholine lipid bilayers: a theoretical and experimental study. RSC Advances. 2015;5(54):43537–46.

[52] Wu J, Liu Q, Yu K, Hu J, Kuo YP, Segerberg M, et al. Roles of nicotinic acetylcholine receptor beta subunits in function of human alpha4-containing nicotinic receptors. J Physiol. 2006;576(Pt 1):103–18. 10.1113/jphysiol.2006.114645 16825297 PMC1995635

[53] Kuo YP, Xu L, Eaton JB, Zhao L, Wu J, Lukas RJ. Roles for nicotinic acetylcholine receptor subunit large cytoplasmic loop sequences in receptor expression and function. J Pharmacol Exp Ther. 2005;314(1):455–66. 10.1124/jpet.105.084954 15833891

[54] Utkin YN. Aging Affects Nicotinic Acetylcholine Receptors in Brain. Cent Nerv Syst Agents Med Chem. 2019;19(2):119–24. 10.2174/1871524919666190320102834 30894113

[55] Hoskin JL, Al-Hasan Y, Sabbagh MN. Nicotinic Acetylcholine Receptor Agonists for the Treatment of Alzheimer’s Dementia: An Update. Nicotine Tob Res. 2019;21(3):370–6. 10.1093/ntr/nty116 30137524 PMC6379052

[56] Lassi G, Taylor AE, Timpson NJ, Kenny PJ, Mather RJ, Eisen T, et al. The CHRNA5-A3-B4 Gene Cluster and Smoking: From Discovery to Therapeutics. Trends Neurosci. 2016;39(12):851–61. 10.1016/j.tins.2016.10.005 27871728 PMC5152594

[57] Liu W, Li MD. Insights Into Nicotinic Receptor Signaling in Nicotine Addiction: Implications for Prevention and Treatment. Curr Neuropharmacol. 2018;16(4):350–70. 10.2174/1570159X15666170801103009 28762314 PMC6018190

[58] Quik M, Boyd JT, Bordia T, Perez X. Potential Therapeutic Application for Nicotinic Receptor Drugs in Movement Disorders. Nicotine Tob Res. 2019;21(3):357–69. 10.1093/ntr/nty063 30137517 PMC6379038

